# Engineered MspA Nanopores for Real-Time Detection of Insulin, Its Pharmaceutical Analogues, and Degradation Products

**DOI:** 10.1021/acsnano.6c10533

**Published:** 2026-10-07

**Authors:** Sabine Straathof, Matthijs Tadema, Lijian Zhan, Giovanni Maglia

**Affiliations:** Department of Chemical Biology, Groningen Biomolecular Sciences & Biotechnology Institute, 3647 University of Groningen, 9747 AG Groningen, The Netherlands

**Keywords:** insulin, MspA, continuous biosensing, artificial pancreas, nanopore, machine learning

## Abstract

Nanopore sensors have demonstrated single-molecule resolution for nucleic acids and small molecules, yet the discrimination and detection of folded proteins in complex biological matrices remains a challenge. Insulin is a demanding test case: it circulates in blood at picomolar concentrations, differs from clinically used pharmaceutical analogues by as little as a single amino acid, and its continuous monitoring would close the remaining open loop in artificial pancreas systems. Here, we show targeted mutations near MspA nanopore’s constriction-enabled discrimination of insulin from the pharmaceutical analogues insulin aspart—differing by one amino acids, and insulin glargine. Furthermore, we reported on insulin degradation by interstrand disulfide reduction and deamidation, a ubiquitous degradation pathway in protein pharmaceuticals that compromises both potency and safety. A machine-learning classifier built on 16 single-event features separated insulin from the blood background with a per-pore limit of detection of 16 nM in 25% v/v blood, suggesting that an array of ∼1000 pores could reach physiologically relevant concentrations. A single-molecule electrical readout for insulin is a requirement for wearable sensors for making an artificial pancreas and could be used for preadministration quality control of insulin pharmaceuticals.

## Graphical abstract

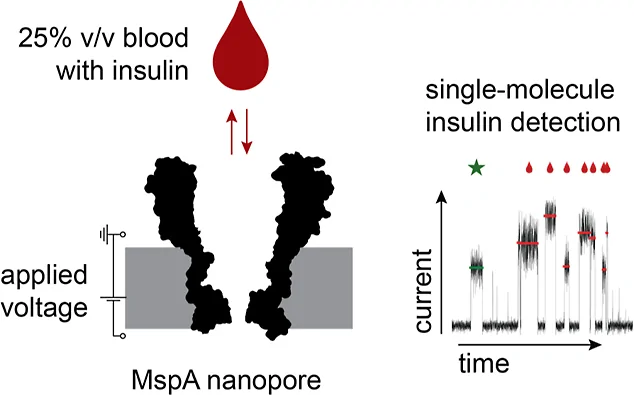

Continuous glucose monitoring^1–5^ is now routine for the >500 million people living with diabetes^6^ and, when coupled with insulin pump systems,^7–15^ has dramatically improved diabetes treatment. However, because of the variability of insulin pharmacokinetics,^16^ continuous monitoring of insulin in the bloodstream remains the missing component needed to achieve an artificial pancreas. Nonetheless, neither a wearable nor even a home-diagnostic insulin sensor yet exists. Insulin circulates at picomolar concentrations (15–100 pM in the fasting state,^17–20^ up to 900 pM postprandially^21,22^ ) in a matrix containing many other proteins at much higher abundance. Immunoassays and mass spectrometry can quantify insulin, but they suffer from antibody cross-reactivity^23–25^ or require extensive sample preparation,^26–28^ neither of which translates readily into a continuous, integrated device. Insulin management is also more demanding than concentration measurement alone: pharmaceutical analogues such as insulin aspart and insulin glargine differ from human insulin by as little as a single amino acid yet have very different pharmacokinetics, and degraded insulin (deamidated or reduced) has reduced bioactivity but is rarely flagged by conventional methods.

Biological nanopores convert single-molecule capture events into ionic-current blockades. Over the past decade, nanopore analysis has been transitioning from a DNA-sequencing platform into a generic single-molecule biosensor. Most of the work focused on small molecules because they are at typically high concentrations and they can passively diffuse across nanopores, with advances in the miniaturization of the nanopore probe allowing the *in vivo* analysis inside living cells.^29^ Two recent advances make proteins plausible targets for nanopore analysis. First, electro-osmotic flow (EOF) can be used to capture polypeptides largely independently of their net charge,^30–33^ removing the main obstacle to detecting weakly charged proteins such as insulin. Second, block copolymer membranes substantially extend bilayer lifetime in whole blood, enabling the first real-time detection of a specific protein from 25% v/v blood.^34,35^

Among engineered nanopores, α-helical ClyA^30,36^ from *Salmonella typhi*, PlyAB^37,38^ from *Pleurotus ostreatus*, and YaxAB^39–41^ from *Yersinia enterocolitica* are typically used to measure large proteins (>20 kDa). β-Barrel MspA from *Mycobacterium smegmatis* and α-helical FraC from the sea anemone *Actinia fragacea* have geometries (>5 nm *cis* opening, ∼1.2/1.6 nm constriction) that allow them to trap and read small proteins such as insulin. MspA-M2, a version engineered for DNA-sequencing applications, has been used to detect ubiquitin (8.6 kDa, 0e),^42^ calmodulin (17 kDa, −24e),^43^ lysozyme (14 kDa, +8e),^44^ and myoglobin (17 kDa, 0e),^44^ but the capture of weakly charged proteins by MspA-M2 is impractical because of the low capture frequency (e.g., ∼15 mM^–1^·s^–1^ for ubiquitin^42^), requiring asymmetric salt buffers to enhance EOF.^44–46^ FraC has a strong EOF that enables detection of macroglobulin (11.6 kDa) and chymotrypsin (25 kDa),^32^ but it cannot be easily integrated into block copolymer membranes. Other nanopores such as α-hemolysin and aerolysin have been used for detecting unfolded polypeptides^47–49^ or DNA.^50–52^ However, their small entry (<2 nm) making them a less obvious choice for the detection of folded proteins, as also highlighted in recent reviews.^36,53–58^

In this study, we demonstrate that MspA-M2 nanopores can capture insulin, albeit with low resolution and infrequent blockades. Mutations that enhance electro-osmotic flow increased the capture frequency and lowered the threshold for capture; however, only the introduction of a charge near the insulin residence site produced blockades that enabled discrimination of human insulin from insulin aspart (which differs by one amino acid) and insulin glargine, indicating that interactions between the protein analyte and the nanopore are important for molecular recognition. Using these engineered nanopores, insulin degradation by deamidation and disulfide reduction—tests used to verify insulin stability, shelf life, and biological potency—could also be measured and quantified. The engineered nanopores exhibited a rich electrical signature, enabling a machine-learning classifier based on 16 single-event features to separate insulin from the blood background. The classifier achieved a per-pore limit of detection of 16 nM in 25% v/v blood. This work describes a single-molecule readout for insulin that is could be used in wearable sensors required for an artificial pancreas.

## Results

Human insulin (5808 Da, −2e) consists of two chains (A and B) that are interconnected with two disulfide bridges (Figures 1A and S1). To detect insulin, we used MspA, a nanopore of compatible size (Figure 1B), and capable of measuring polypeptides ∼2–15 kDa in size.^44,59^ The entry of molecules into the nanopore is facilitated by the electro-osmotic flow (EOF),^60–62^ while electrostatic interactions between the pore and the protein might influence the residence of proteins inside the nanopore. Therefore, we selected three MspA nanopores with different ion selectivity: cation-selective MspA-D90N (*P*_Cl^–^_/*P*_K^+^_ = ∼0.14 from ref 63), slightly anion-selective, and highly cation-selective MspA-M2-S103K–N108K and MspA-M2-S103K–I105K (*P*_Cl^–^_/*P*_K^+^_ = 2.64 and ∼3.99, respectively;^60^ Figure 1C, with MspA-M2 including the D90N-D91N-D93N-E118R-D134R-E139K, mutations).

**Figure 1:**
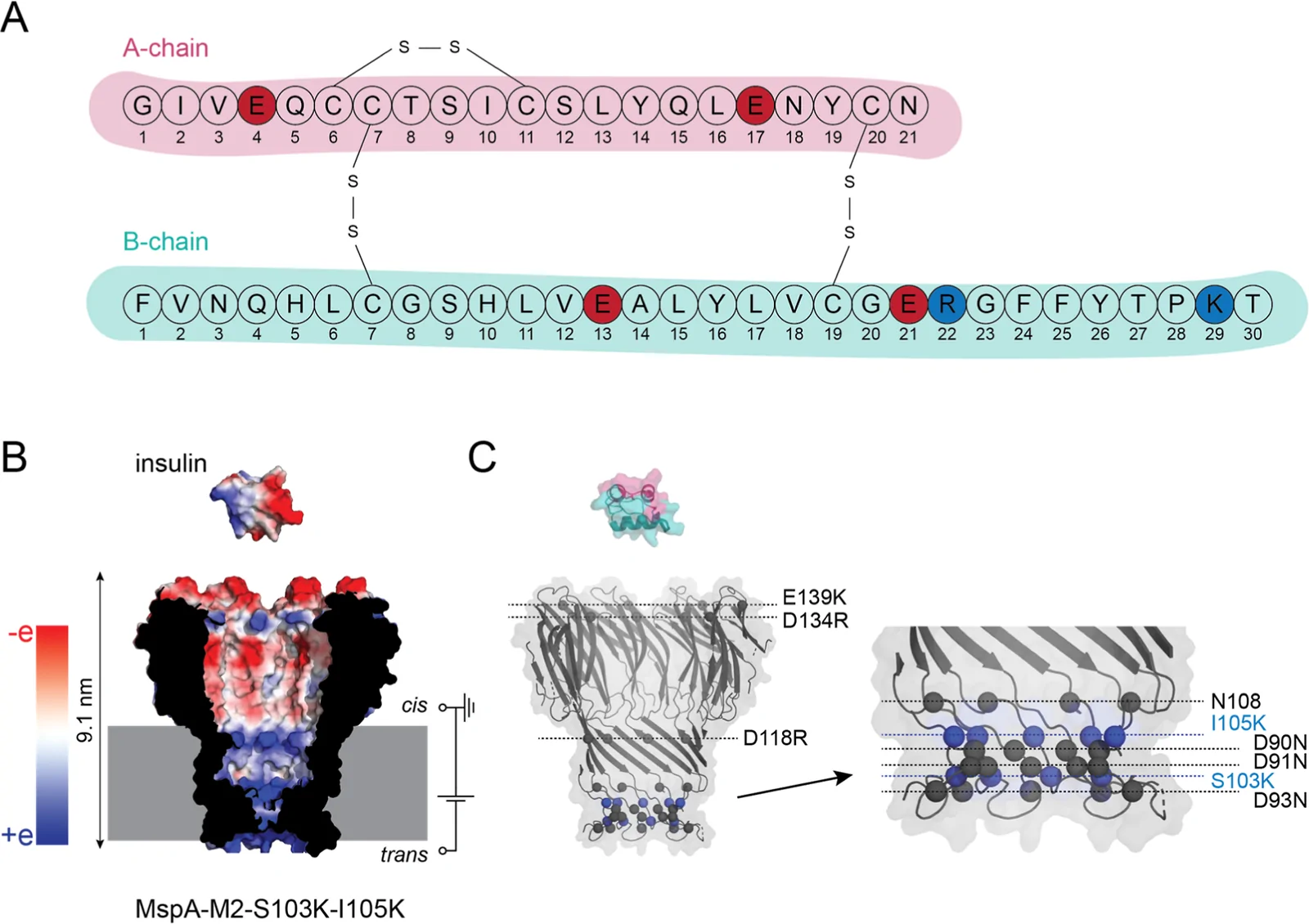
Insulin and MspA. (A) Primary amino acid sequence of insulin. A-chain is pink and B-chain is in teal. Negative residues (aspartate D and glutamate E) are in red and positive residues (lysine K and arginine R) are in blue. Disulfide bridges between cysteines (C) are indicated by S–S. (B) Electrostatic map of insulin and cross-section of MspA-M2-S103K–I105K. Cross-section surface is in black. Bilayer is in gray. Proteins are to scale. (C) Cartoon representation of insulin and cross-section of MspA-M2-S103K–I105K. *z*-position of the mutated residues are indicated by the α-carbon, with S103K and I105K in blue. Images were generated in PyMOL.

The addition of human insulin (*cis*, 1 M NaCl, 50 mM Tris–HCl pH 7.5) to the MspA nanopores induced blockades (Figure 2B) at positive applied potential for anion-selective MspA nanopores and at negative applied potentials for cation-selective MspA-D90N (Figures S2–S4). Protein blockades were categorized for their excluded current *I*_EX_ [%] = [(*I*_O_ – *I*_B_)/*I*_O_] × 100% and noise level σ_blockade_[pA].

**Figure 2:**
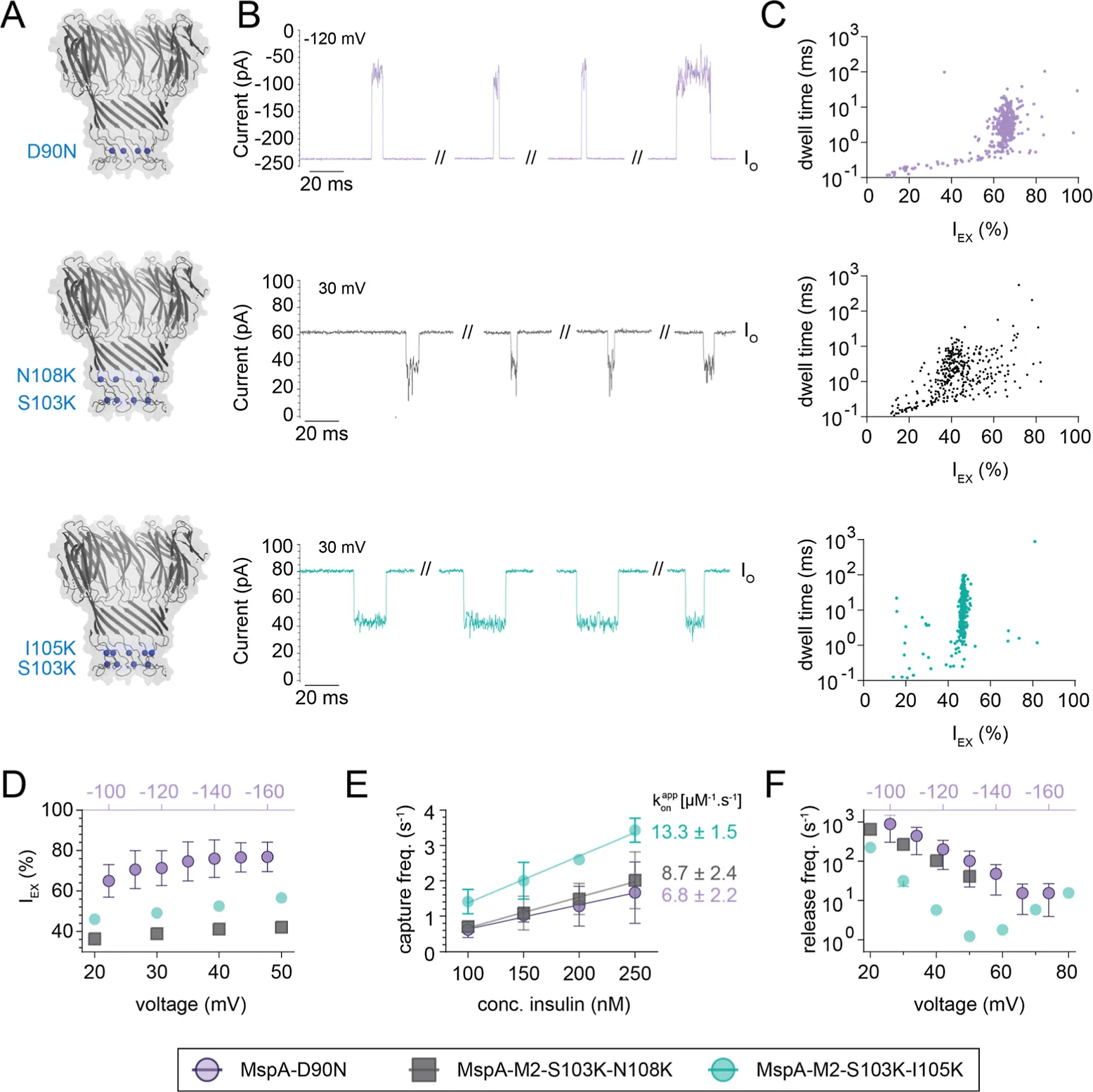
Human insulin detection by MspA mutants. (A) Cross-section of MspA indicating α-carbon D90N, S103K, I105K, and N108K in blue. (B) Electrophysiology blockades of human insulin by the respective MspA mutant. Open pore current (*I*_O_) is indicated. (C) Scatterplots of insulin blockades, *n* = 400 events per pore. Insulin was added to *cis* at 100 nM. Measurements were conducted at +30 mV. (D) Excluded current (*I*_EX_) and (E) release frequency of human insulin at increasing voltage (250 nM, *cis*). (F) Capture frequency of human insulin at increasing concentration. The apparent *k*_on_-values indicated (mean μ ± standard error of the mean SEM) were obtained by least-squared linear regression analysis in GraphPad 9.5.0. Measurements were conducted in 1 M NaCl, 50 mM TrisHCl pH 7.5. Data were recorded at 50 kHz sampling rate and 10 kHz Bessel filter. Traces were additionally filtered with 2 kHz low-pass Gaussian filter for analysis and visualization. Experiments were conducted at minimum in triplicate (*N* > 3 pores). Data in (D, E, and F) are shown as mean ± SD. Error bars are smaller than the symbol size and therefore not visible.

Insulin blockades in MspA-M2-S103K–I105K and MspA-D90N often displayed a secondary, deeper level (L2, Figures S3 and S4), which became more predominant at higher applied potentials. Blockades with two levels are often seen in nanopore experiments with a variety of analytes, including proteins.^64–66^ A likely explanation is that as the applied voltage is increased, insulin is pulled closer into the MspA constriction (residue 90 and 91) reducing the flow of ion current. The secondary insulin levels were not observed for MspA-M2-S103K–N108K (Figure S5), albeit voltages higher than +50 mV could not be tested because of the gating of the nanopore (Figure S6). These voltage-dependent blockades provide useful parameters to distinguish proteins in complex biological samples.

MspA-M2-S103K–I105K (+30 mV), MspA-M2-S103K–N108K (+30 mV), and MspA-D90N (−120 mV) captured insulin with *I*_EX_ of 48.5 ± 0.2%, 39.1 ± 0.1% and 71.7 ± 2.2%, respectively, and σ_blockade_ = 3.3 ± 0.1 pA, 4.6 ± 0.1 pA, and 17.4 ± 1.0 pA, respectively (Figures 2C and S7). The apparent on-rates (*k*_on_^app^) of MspA-M2-S103K–I105K, MspA-M2-S103K–N108K, and MspA-D90N were 13.3 ± 1.5 μM^–1^ s^–1^, 8.7 ± 2.4 μM^–1^ s^–1^, and 6.8 ± 2.2 μM^–1^ s^–1^, respectively; Figures 2 and S7).

The apparent off-rate (*k*_off_^app^, inverse of dwell time, s^–1^) of insulin in MspA-D90N and MspA-M2-S103K–N108K decreased throughout the voltage range sampled, suggesting that insulin did not translocate the nanopore (Figures S8 and S9). By contrast, MspA-M2-S103K–I105K initially decreased over voltage to then increase at voltages higher than +50 mV (Figures 2F and S10), a hallmark of translocation.^62^

Collectively, MspA-M2-S103K–I105K captured insulin at a higher rate with larger and more specific blockade compared to MspA-M2-S103K–N108K and MspA-D90N. Furthermore, compared to MspA-D90N, MspA-M2-S103K–I105K captured insulin at a lower applied potential. This is advantageous for sampling proteins in complex biological samples in which multiple components might enter and block the nanopore.

### Identification of Insulin Analogues

Diabetes patients are often prescribed pharmaceutical insulin analogues for personalized glycemic control. The fast-acting insulin aspart is biologically active within 15 min and for up to 10 h after administration, whereas long-acting insulin glargine can be biologically active within 2–4 h and up to 24 h after administration.^67^ Insulin aspart (5826 Da, −3e) has mutated proline 28 of the B-chain into aspartate (P28D, Figures 3A and S1) resulting in an additional negative charge at physiological pH (Table S2). Insulin glargine (6051 Da, 0e) has mutated asparagine 21 of the A-chain into glycine (N21G) as well as two additional arginines on the B-chain (R31–R32), resulting in a net charge of 0e at physiological pH (Figure 3A and Figure S1 and Table S2).

**Figure 3:**
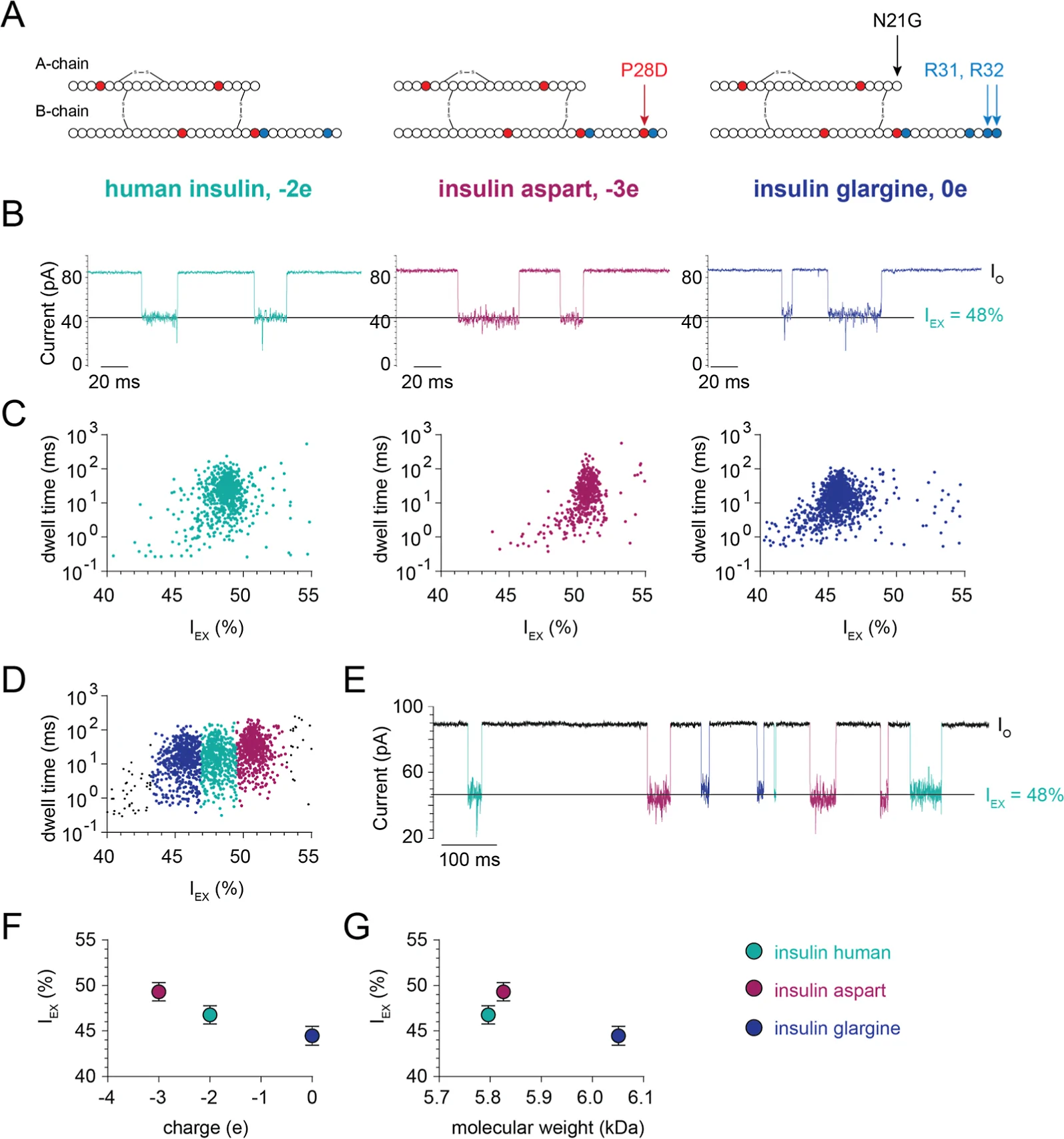
Insulin analogue detection by MspA-M2-S103K–I105K. (A) Schematic representation of human insulin (teal), insulin aspart (burgundy), and insulin glargine (dark blue). Negative residues (aspartate D and glutamate E) are in red, positive residues (lysine K and arginine R) are in cyan. (B) Current blockades of corresponding insulin analogues as detected by MspA-M2-S103K–I105K. (C) Scatterplots of insulin and its analogues, with *n* > 500 events for each protein. Human insulin and insulin aspart were, respectively, added to *cis* at 100 nM. Insulin glargine was added to *cis* at 114 nM. (D) Scatterplot and (E) current trace of mixed solutions of human insulin, insulin aspart, and insulin glargine at equimolar ratio (250 nM each in *cis*). (F) Charge and (G) molecular weight versus *I*_EX_ for three insulin analogues in the mixture. Error bars in F and G indicate standard deviation. Measurements were conducted at +30 mV, in 1 M NaCl, 50 mM TrisHCl pH 7.5. Data were recorded at 50 kHz sampling frequency and 10 kHz Bessel filter. Traces were additionally filtered with 2 kHz low-pass Gaussian filter for analysis and visualization. Experiments were conducted at minimum in triplicate (*N* > 3 pores).

Insulin assays (e.g., ELISAs), which might be used for managing diabetes, can determine the insulin concentration in blood with high sensitivity but often fail to discriminate between insulin analogues due to cross-reactivity of the insulin-binding antibodies.^68–70^ HPLC-MS and derived techniques are able to discriminate insulin analogues at good sensitivity but need excessive sample preparation (e.g., precipitation/centrifugation or immuno-/aptamer-enrichment) before measuring blood samples.^71–76^

The three insulin analogues were prepared as an equimolar mix (250 nM) and added to the *cis* side of MspA nanopores (*n* > 500 events, Table S3). MspA-M2 could distinguish between insulin and insulin aspart, but the blockades between insulin and insulin glargine largely overlapped (Figure S11). MspA-M2-S103K–N108K showed overlapping blockades for the three insulin analogues (Figure 12). By contrast, MspA-M2-S103K–I105K showed a good separation for all the three insulin analogues at bias +20 to +50 mV (Figures 3D,E and S13).

Insulin analogs were tested individually with MspA-M2-S103K–I105K nanopores. The blockades confirmed a different excluded current (*I*_EX_ = 48.5 ± 0.2%; 52.0 ± 0.3%, and 44.4 ± 0.2% at +30 mV, for human insulin, insulin aspart, and insulin glargine, respectively, Figure 3B,C). The respective capture and release frequencies also slightly differed, while the current noise (σ_blockade_) was similar (Figure S14). Upon increasing voltage, insulin aspart translocated at *V* > +45 mV (Figure S15), whereas insulin glargine did not translocate (Figure S16). Interestingly, the *I*_EX_ of insulin and its analogues correlated well with their net charge at physiological pH (Figure 3F) and poorly with their molecular weight (Figure 3G). This suggests that the protein signal is likely influenced by the excluded volume and by the position relative to the constriction, as also observed with larger conical nanopore YaxAB.^41^

To explore the molecular bases of insulin recognition, we tested additional individual 103 and 105 substitutions. Position 105 is above the constriction (i.e., on the *cis*-side), which is formed by residues at positions 90 and 91 (Figure 1C), whereas position 103 is below the constriction (i.e., *trans*-side; Figure 1C). MspA-M2-I105K (*P*_Cl^–^_/*P*_K^+^_ = 2.21 from ref 60) showed overlapping distributions (Figure S17). Alternatively, MspA-M2-S103K (*P*_Cl^–^_/*P*_K^+^_ = 2.44 ^60^) showed a good separation, but the bias had to be increased to above +80 mV (Figure S18). This suggests that, most likely, a strong EOF is beneficial to bring insulin near the constriction of the nanopore, while a positive charge near the residence site of the protein within the nanopore allows us to distinguish between the different insulin analogues.

### Detection of Insulin Degradation for Quality Assessment

In proteins, asparagine residues can undergo deamidation, a nonenzymatic, spontaneous post-translational modification (PTM), in which an asparagine side chain (114.10 Da) is converted into an aspartate side chain (115.08 Da) through a succinimide intermediate (Figure 4A). Glutamines can also deamidate—into glutamate—although it occurs less frequently.^77^

**Figure 4:**
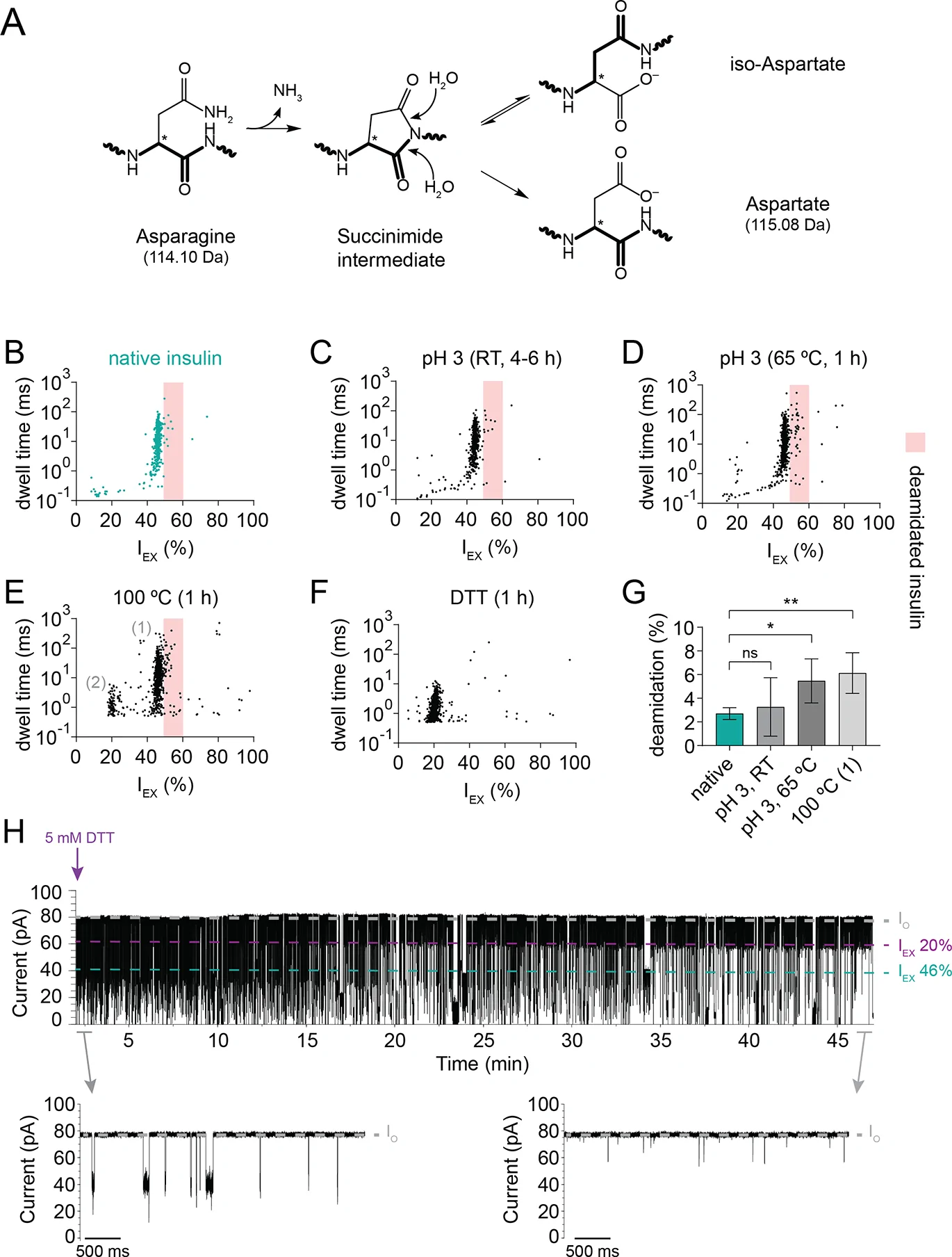
Insulin degradation detection by MspA-M2-S103K–I105K. (A) Asparagine deamidation scheme. Protein backbone is in bold, α-carbon is indicated with asterisk (*). (B) Scatterplot of untreated, native human insulin. Red block indicates expected deaminated insulin blockades (*I*_EX_ 48.9–60.0%). (C) Scatterplot of human insulin preincubated at pH 3 (50 mM acetic acid, 4–6 h at room temperature). (D) Scatterplot of human insulin preincubated at pH 3.0 and at 65 °C (50 mM acetic acid, 1 h). (E) Scatterplot of human insulin preincubated at 100 °C (1 h). Cluster at *I*_EX_ ∼ 46% is indicated as (1), cluster at *I*_EX_ ∼ 20% is indicated as (2), in gray. (F) Scatterplot of human insulin preincubated in 5 mM DTT (1 h, RT). (G) Comparing insulin deamidation ratio after the different treatments. Error bars indicate standard deviation. Experiments were executed at minimum in triplicate (*N* > 3 pores), with *n* > 500 data points per replicate. Treatments were analyzed with an unpaired *t*-test (GraphPad Prism 9.5), resulting in ns (nonsignificant) difference, or significant difference, indicated with an asterisk (* for *p* = 0.01, ** for *p* = 0.003). (H) Continuous electrophysiology trace of 45 min of MspA-M2-S103K–I105K detecting human insulin, while 5 mM DTT (purple arrow) was added at 0 min. Zoom-in of start and end of trace are below. In all experiments, (treated or untreated) human insulin was added to cis at 250 nM. Measurements were conducted at +30 mV, in 1 M NaCl, 50 mM TrisHCl pH 7.5. Data were recorded at 50 kHz sampling frequency, 10 kHz Bessel filter. Traces were additionally filtered with 2 kHz low-pass Gaussian filter for analysis and visualization.

Human insulin contains three asparagine residues (Figure 1A) that can be deamidated. Deamidation results in an additional negative charge in the protein, potentially affecting structure and activity.^78,79^ Since human insulin and insulin aspart differ by one charge and can be distinguished (Δ*I*_EX_ = 3.5 Δ% at +30 mV, 1 M NaCl, Figures 3 and S14), we expect that insulin deamidation should also be observable in MspA-M2-S103K–I105K by an equivalent increase of the *I*_EX_ with ∼3.5 Δ%.

We found that 2.7 ± 0.5% of insulin events (*N* = 5) had an *I*_EX_ value in the current range expected for deamidation (Δ*I*_EX_ range 48.9–60.0%). Analysis using nano-LC-MS on a C18-column confirmed that deamidation of insulin likely occurred, albeit from the area of the native and deamidated peptides, a lower deamidation percentage was expected (∼0.1%, Figure S19). Deamidation shifts peptide mass by just +0.984 Da, almost exactly the mass of the native peptide’s natural ^13^C isotope (M + 1) peak. This near-total overlap is a well-recognized limitation of bottom-up LC–MS/MS for quantifying deamidation, especially at low abundance. Nanopore sensing works on a fundamentally different principle, so the two methods’ absolute percentages are not expected to match precisely. Here, however, the gap is substantial. By reading single molecules directly, nanopore sensing may offer greater sensitivity for rare modifications such as deamidation, which LC–MS/MS struggles to distinguish from the native peptide signal.

Deamidation is dependent on the temperature, pH, and can be influenced by residues flanking the asparagine.^80–82^ Therefore, to accelerate degradation, insulin (10 μM) was preincubated under three conditions: (1) pH 3, at room temperature (RT) for 4–6 h; (2) pH 3, at 65 °C, for 1 h; and (3) pH 7.5, at 100 °C, for 1 h. Samples were then analyzed with MspA-M2-S103K–I105K (250 nM in *cis*, Figures 4B–E and S20) and nano-LC-MS (Figure S19).

The main insulin events revealed no change in *I*_EX_ and σ_blockade_ for all samples (Figure S21). However, some events appeared in the expected deamidation window (Figure S22). The amount of these higher *I*_EX_-events increased to 5.4 ± 1.9% (*p* = 0.014 in unpaired *t*-test, *N* = 4) when insulin was pretreated at 65 °C in pH 3 (Figure 4G). In fact, boiling insulin (pH 7.5, 100 °C) increased the amount of these expected deamidation events to 6.1 ± 1.7% (*p* = 0.003 in the unpaired *t*-test, *N* = 5, Figure 4G). Nano-LC-MS analysis confirmed deamidation, however, a correlation could not be established (Figure S19), most likely reflecting the difficulty of MS to quantify deamidation.

Additional degradation of insulin, not observed by MS, i.e., amount of broken disulfide bridges, was captured by nanopore analysis. Upon boiling insulin at pH 7.5, a secondary cluster appears at *I*_EX_ = 19.5 ± 1.0% (*N* = 5; Figure 4E). Since the signal was at lower *I*_EX_, we theorized it could have been generated by the translocation of individual insulin chains. Preincubated in reducing conditions (5 mM DTT, RT, 1 h), induced a single cluster at a similar *I*_EX_ (20.7 ± 0.5%, *N* = 3; Figures 4F and S23), confirming that the additional signal reflected the translocation of the linearized A- or B-chains of insulin across the nanopore. The reduction of insulin (at 5 mM DTT) can be observed in real time over 45 min (Figure 4H).

### Machine-Learning-Based Insulin Detection from 25% v/v Blood

As a diagnostic tool in the clinic, at home, or in wearable devices, an autonomous analysis workflow is required that can distinguish insulin in real time from other proteins in blood or interstitial fluid. A working concentration of 25% v/v blood was selected as a technically feasible starting point for analyte sensing,^34^ with higher blood fractions targeted in future work.

To discriminate insulin from other proteins in blood with MspA-M2-S103K–I105K, we extracted 16 features from insulin blockades and from blood blockades (recorded at +30 mV, from 25% v/v blood in *cis*, i.e., “blood only”). These features included the mean, median, σ_blockade_, skewness, kurtosis, the means of the high and low gaussians of a Gaussian mixture fit of the *I*_EX_ %, and the natural logarithm of the dwell time (Figure 5A). In addition, we included the power spectral density of the *I*_EX_ and used the eight frequencies with the highest amplitude describing the behavior of the event in the frequency space. The annotated insulin and blood blockade parameters were partially cleaned up to prevent pollution of the data set (see Methods).

**Figure 5:**
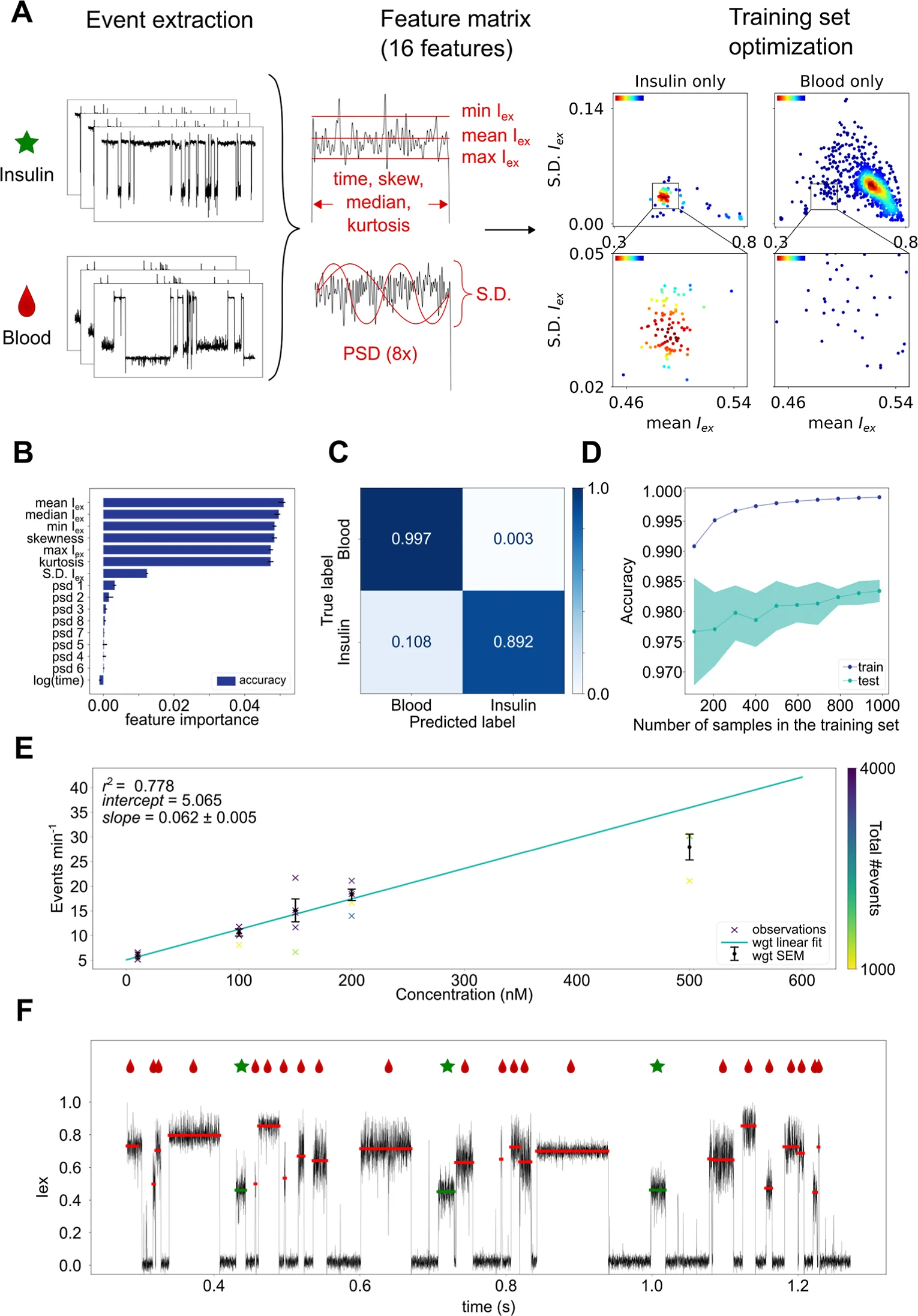
Machine learning-based detection of insulin in 25% v/v blood. (A) Schematic workflow of the machine learning classification model training. Events are extracted from preprocessed electrophysiology traces with nanotrace, and a feature matrix including 16 features per event is built. This training set is optimized by removing outliers (see Methods section). (B) Permutation analysis of the feature matrix indicates event feature importance. (C) Confusion matrix displaying the frequency of true negative, false positive, false negative, and true positive classification of the entire data set (*n* = 15,642). (D) Learning curve showing a classifier accuracy on training and test data as a function of the number of samples in the training set. Shaded area represents the standard deviation in the accuracy. (E) Calibration curve of classified “insulin”-events in blood. Observations (×) are events per minute per experiment. Standard error is the standard error of the mean. The linear regression and error bars are weighted by the total number of events. (F) The annotated electrophysiology trace showing identified insulin events as green stars and events identified as background (red drops). Measurements were conducted in 1 M NaCl, 50 mM TrisHCl pH 7.5. Data were recorded at 50 kHz sampling frequency, 10 kHz Bessel filter. Experiments were conducted at minimum in triplicate (*N* > 3 pores).

These features were used to train a machine learning classifier in a supervised manner. Although machine learning algorithms have been applied in nanopore analysis before,^83–85^ these were mainly employed to identify events with few nontarget contaminations, i.e., many targets and few background events. In our experiments, the opposite is true: a few target events and many background events. The uniformity of insulin events allowed us to treat this task as an outlier detection problem (see Methods).

Briefly, we fitted an elliptic envelope model to the insulin features and tuned the decision threshold (“insulin” or “blood”) for optimal separation. A perturbation analysis revealed that the features describing the distribution of *I*_EX_ values were most informative for decision-making, while the power spectral density frequencies provided significantly less information (Figure 5B). This could be due to a large default window size parameter for the power spectral density calculation using Welch’s method,^86^ resulting in a low resolution in the frequency space. In addition, this analysis pipeline indicated that the dwell time was not a useful feature to distinguish insulin events from the blood background.

The decision threshold was also tuned with an accuracy metric. The confusion matrix indicated that our classifier performed with a low false positive frequency but at the cost of a higher false negative frequency (Figure 5C). While this is good for accuracy, it effectively increased the limit of detection (LOD) slightly because some true insulin events are mislabeled.

The efficiency of the machine learning algorithm was assessed with a learning curve (Figure 5D), which depicts the accuracy as a function of samples in the training set and reports on the potential overfitting of the model. In our model, the training curve plateaus around 1000 samples (from *n* = 1095 insulin events from *N* = 5 pores), while the test accuracy is still increasing. This suggests that our classifier could make better predictions when given more data until the full plateau is reached. The false-positive rate of our model was 0.3% (Figure 5C). This means that, in blood-only data, about 44 out of 14,547 events were classified as insulin.

Next, we employed MspA-M2-S103K–I105K to measure insulin (10–500 nM, *cis*) in a background of blood (25% v/v, *cis*) at +30 mV and used the tuned model to predict the class of each event (“insulin” or “blood”). The number of events was normalized for the total measuring time of the independent measurement and was plotted against the concentration (Figure 5E). We used a weighted linear fit (*R* ^2^ = 0.778), weighted by the number of events in each experiment, to determine the k_on_^app^ at 0.062 ± 0.005 nM^–1^ min^–1^ (1.03 ± 0.08 μM^–1^ s^–1^; errors indicating standard error).

So, the insulin capture in background of 25% v/v blood is reduced by 13-fold compared to measurement in buffer (Figure 2F), thereby providing a quantification of the “contamination” of blood proteins. Notably, Fahie et al. observed 3-fold reduction in on-rate of functionalized OmpG in 10% v/v serum compared to buffer experiments,^87^ where the quantification was not perturbed by serum blockades, suggesting that a reduction of on-rate in a crowded environment is to be expected. When we arbitrarily define the LOD at 1 classified event per minute (an illustrative benchmark of system sensitivity rather than a formally validated analytical LOD), the LOD would be at 16.1 nM, which is about a 1000-fold too low to measure physiological relevant concentrations using a single nanopore. An array of 1000 nanopores should allow the detection of physiological concentration of insulin directly in blood.

## Discussion and Conclusion

In this study, we characterized insulin and its pharmaceutical analogues using engineered MspA nanopores with an enhanced electro-osmotic flow. Although insulin could be captured by several nanopore variants, only the anion-selective MspA-M2-S103K and MspA-M2-S103K–I105K pores reliably distinguished human insulin from the pharmaceutical analogues insulin aspart and insulin glargine. Other nanopores, including MspA-M2-S103K–N108K, MspA-M2-I105K, and MspA-M2, showed markedly lower discriminatory power.

These results highlight the crucial role of the constriction in detecting molecular differences. An anion-selective nanopore is required for an efficient capture and residence time of the protein inside the nanopore. However, the residence of proteins inside the nanopore is *per se* not sufficient for observing molecular differences in proteins. Efficient separation from insulin analogues—with insulin and insulin aspart differing by a single charge (P28D)—was only observed when lysine residues were introduced at position 103 or at positions 103 and 105, which are located near the constriction of the nanopore. By contrast, when lysine residues were introduced closer to the *cis*-entry at position 108 (or when MspA-M2 was used), the resolution was greatly reduced. A likely explanation is that molecular recognition is associated when proteins are residing near the constriction of the nanopore. The latter is favored by a strong EOF and affected by the charge of the protein and by the interaction between the protein and the nanopore surface. The latter allows one to fine-tune the resolution of the nanopore for molecular differences.

MspA-M2-S103K–I105K was also used to identify insulin degradation by deamidation or disruption of interstrand disulfide linkages, two degradation pathways that are accelerated by heat and pH.^80,82^ Deamidation is typically measured by LC–MS. However, quantitative analysis is challenging because deamidation can be artifactually induced during sample preparation. Here, we found that the charge change in deamidated of insulin likely shifts the excluded-current distribution (Δ*I*_EX_ ≈ + 3.5%), allowing this population to be quantified. Therefore, a nanopore sensor could be developed for quality control during insulin production or to check insulin quality before administration with no or minimal sample preparation step.

A real-time sensor for insulin in blood is the last missing piece for an artificial pancreas. We found that the signal of insulin using MspA-M2-S103K–I105K was relatively complex (i.e., several substates were observed), which facilitates its recognition in a complex biological sample. A machine-learning classifier built on 16 single-event features could correctly identify 89% of insulin events with a per-pore limit of detection of 16 nM in 25% v/v blood. The signal variations in the current blockade—likely to arise from fast motion of the proteins^64–66^ as the analyte interacts with the inner nanopore surface—provided additional discriminatory features that improved classification. However, the LOD is ∼18-fold above the postprandial range (∼900 pM) and 160–1000-fold above fasting concentrations (15–100 pM),^88–92^ so a single-pore wearable is not yet realistic.

MspA’s apparent on-rate for insulin in buffer (∼10 μM^–1^ s^–1^) is slightly lower than that of other nanopores,^41,93–96^ and it drops a further 13-fold in 25% v/v blood as abundant matrix proteins compete for access to the pore lumen. Improving capture frequency may require nanopores with a wider entry. Approaches to limit the entry of highly abundant proteins into the nanopore have been devised by engineering a molecular sieve^93^ or by introducing disordered polypeptides above the nanopore together with specific binders for the target protein.^34^ Furthermore, the classifier’s false-negative rate inflates the effective LOD, so additional training data should improve recovery without sacrificing the low false-positive rate. Detection of fasting-range insulin concentrations, however, should also be plausible with the current nanopores by using parallelized nanopore arrays,^97–99^ where the combined event rate scales with pore count. Devices offering 10^3^ to 10^4^ pores on a single chip are already available.

Taken together, these results indicate that engineered MspA nanopores could provide a suitable platform for the electrical readout of insulin in blood when they are implemented in nanopore arrays. Devices based on individual nanopores may also prove to be useful for distinguishing insulin analogues or monitoring insulin degradation in real time.

## Methods

### Materials

Diphytanoyl-*sn*-glycero-3-phosphocholine (DPhPC, CAS #207131-40-6), hexadecane 99% (CAS #544-76-3), insulin human (CAS #11061-68-0, catalog no. I0908), insulin aspart (European Pharmacopoeia (EP) Reference Standard; Y0000349), and insulin glargine (EP Reference Standard; CAS #160337-95-1) were ordered from Sigma/Merck. Defibrinated sheep blood (catalog no. SR0051B, article no. 12967755, Establishment Approval Number 03/215/8002) was purchased from Thermo Fisher Scientific. GeneJET Plasmid Miniprep Kit (K0503), Low Protein Binding Microcentrifuge Tubes (1.5 mL, 15,352,617), and insulin-like growth factor 1 (IGF-1; 17,869,932) were ordered from Thermo Fisher Scientific. Proinsulin (HY-P702540) was ordered from Bioconnect. (Poly)­butadiene-*b*-ethylene oxide (PDB_11_PEO_8_, 650-*b*-350 g/mol, P41807C-BdEO) was ordered from Polymer Source, Inc. Other chemicals used were ordered from Carl Roth. Sequencing was performed by Macrogen and Eurofins. DNA primers were ordered from Integrated DNA Technologies (IDT).

### Cloning of MspA Variants

Plasmids containing MspA mutants were kindly provided by Dr. A. Sauciuc. An overview of the mutant protein sequences can be found in Table S1.

### Protein Purification and Oligomerization

Protein purification was performed as described previously in ref 100.

### Electrophysiology Recordings in Planar Bilayers

An electrophysiology chamber composed of two 500 μL compartments (*cis* and *trans*) separated by a 20 μm PTFE film with a central aperture of ∼100 μm was used for all experiments. To form a bilayer, a drop of hexadecane (4% v/v in pentane) was placed on the aperture on the *trans* side of the PTFE film and allowed to evaporate for ∼2 min. Each compartment was then filled with 400 μL electrophysiology buffer (1 M NaCl, 50 mM TrisHCl pH 7.5), and two drops of lipids (DPhPC, 5 mg/mL w/v in pentane) or block copolymers (PDB_11_PEO_8_, 5 mg/mL w/v in pentane) were added. Ag/AgCl electrodes were inserted into each compartment: *trans* served as the working electrode and *cis* as the ground electrode. By lowering and raising the buffer level in one compartment above the aperture, a bilayer could be formed. After 5–10 min, nanopores were added to the *cis* solution. Before use, purified MspA pores were diluted 500–100× in buffer (150 mM NaCl, 50 mM TrisHCl pH 7.5, 0.02% DDM). Then, a small volume (<0.1–0.3 μL) of appropriately diluted MspA pores was added to the *cis* chamber.

Protein (analyte) concentration was determined by the Lowry assay or by spectrophotometric absorbance at 280 nm. Analytes were added to the *cis* chamber. Pore conductances varied only marginally between DPhPC and PDB_11_PEO_8_ (Figure S24), and we therefore did not distinguish between membrane compositions during protein-capture experiments in buffer. By contrast, experiments conducted in blood were always performed in membranes composed of PDB_11_PEO_8_ because lipids are unstable in blood.^34,41^ Experiments were performed at least in triplicate. Between blood experiments, the chamber was briefly (∼10 min) incubated with 1 M NaOH to remove residual protein. Measurements were conducted at a 50 kHz sampling frequency with a 10 kHz Bessel filter, unless otherwise specified.

### Electrophysiology Data Recording and Analysis

All experimental nanopore data for proteins were recorded under a negative applied potential (*trans*) for cation-selective pores and a positive applied potential for anion-selective pores. Data were recorded using an Axopatch 200B patch-clamp amplifier connected to a DigiData 1440 A/D converter (Axon Instruments) and Clampex 10.7 software (Molecular Devices). *I*/*V* curves were measured from −100 to +100 mV in increments of 10 mV. Recordings were acquired either in the gap-free mode or using a sweep protocol (−20 mV for 250 ms, 0 mV for 250 ms, +30 mV for 1 s; 600 sweeps corresponding to 10 min of recording time). Recordings were analyzed with Clampfit 10.7 software (Molecular Devices). Data were digitally filtered with a Gaussian low-pass filter with a 2000 Hz cutoff before analysis. *I*_O_ was determined by the Gaussian fitting of the all-points histogram with a bin width of 0.5 pA. Protein blockades (*I*_B_), dwell time, and interevent time were detected using the Single-Channel Search function in Event Detection. Event detection was monitored manually. The excluded current percentage (*I*_EX_ [%]) was calculated as [((*I*_O_ – *I*_B_)/*I*_O_) × 100%] for all events using an in-house MATLAB script (script #1).

For experiments carried out in buffered solution, the average dwell time (τ_off_) and average interevent time (τ_on_) were calculated using an in-house MATLAB script (script #1) by plotting the respective dwell-time or interevent-time distributions of at least 500 events as cumulative histograms (edges: 0–5000 ms), to which a standard exponential was fitted. For experiments with MspA-D90N, at least 112 events were used. The release frequency (*f*_R_ = 1/τ_off_, s^–1^) is equal to the off-rate (*k*_off_, s^–1^), i.e., *k*_off_ = 1/τ_off_. The capture frequency (*f*_C_ = 1/τ_on_, s^–1^) was converted to the on-rate (*k*_on_, M^–1^ s^–1^) according to *f*_C_ = *k*_on_ × [POI], where [POI] is the concentration of the protein of interest added to *cis*. The average on-rate per POI was calculated by least-squares regression using GraphPad Prism 9.5.

### Modified Insulin Detection Experiments

In this set of experiments, the pores were slightly smaller (2.6 ± 0.1 nS at +30 mV) due to a new PDB_11_PEO_8_ batch and resulting in slightly smaller insulin blockades (*I*_EX_ = 46.1 ± 0.9%, *N* = 5 at +30 mV). We therefore set the expected deamidation window at minimum *I*_EX_ + 3σ (48.9%) and at maximum *I*_EX_ = 60%.

Human insulin (10 μM, 100 μL) was preincubated under the following conditions: (1) 5 mM DTT, RT, 1 h; (2) 50 mM acetic acid, pH 3, RT, 4–6 h; (3) 50 mM acetic acid, pH 3, 65 °C, 1 h; and (4) 100 °C, 1 h. After preincubation, the samples were briefly spun down (5000 rpm for 3 s) to remove condensate from the lid. The samples were then placed on ice to quench the reaction, except for condition 2 (50 mM acetic acid, pH 3, RT, 4–6 h), which was kept at RT. Samples were measured on the same day as preincubation, except for condition 4 (100 °C, 1 h), measured over the following 2 days after overnight storage at 4 °C.

### Mass Spectrometry

For each insulin sample, treated as described above, ∼200 pmol was taken for LC-MS-based peptide analysis. Samples were first reduced with 10 mM TCEP at 37 °C for 60 min with agitation, and the resulting thiol groups of cysteine residues were then alkylated by incubation with 25 mM iodoacetamide in the dark for 30 min. The samples were acidified by addition of formic acid to a final concentration of 0.1% (v/v). The samples were then analyzed by nanoflow HPLC (Ultimate 3000 RSLC, Thermo Fisher) coupled to a tandem mass spectrometry system (Exploris 480, Thermo Fisher). For each sample, 1 μL (5 pmol) was loaded onto a C18 trap cartridge (Acclaim PepMap C18 Reversed Phase Trap Cartridge, 5 μ, 0.3 mm I.D. × 5 mm L., Thermo Fisher) and then separated by reverse-phase chromatography using a 75 μm I.D. × 40 cm L. nano-LC column (PepSep C18 Repro-Sil AQ 1.9 μm, Bruker). Peptides were eluted with a linear gradient from 95% solvent A (0.1% FA) and 5% solvent B (80% acetonitrile, 0.1% FA) to 35% solvent B over 30 min at a flow rate of 300 nL/min at 45 °C. Mass analysis of the eluent was performed online using nanoelectrospray ionization. The Data-dependent acquisition (DDA) mode was used to obtain high-resolution master scans from 385 *m*/*z* to 1540 *m*/*z* at a resolution of 120,000 at *m*/*z* 200. Ions with charge states from +2 to +6 were selected for MS/MS analyses, whereas ions with unassigned charge states were excluded. The selected ions were further sorted according to abundance and fragmented by HCD at 30% normalized collision energy; the resulting fragment ions were then measured by data-dependent MS2 scans at a resolution of 15,000. Dynamic exclusion for the same precursor ions was set to 20 s, and ion accumulation time was set to auto for both master scans and ddMS2 scans. The cycle time between two master scans was set to 2 s. Additional treatments (37 °C for 70 h, pH 9.5, and pH 3) were applied exclusively for mass spectrometry analysis and were not used in any other measurements. Raw data were analyzed using PEAKS Studio 12.5 software (Bioinformatics Solutions) by database searching against the insulin sequence. Carbamidomethylation and half-disulfide bridges at cysteines, oxidation of methionines, deamidation of asparagines and glutamines, and N-terminal acetylation were set as variable modifications. Mass tolerances were set to 10 ppm for precursor ions and 0.02 Da for fragment ions. Proteins were reported when peptide FDR and protein FDR were both <1%. Modifications with a PTM Ascore <15 were filtered out. Modified and unmodified peptides were then compared by the peak area under the XIC curves, and the resulting ratio was used to calculate the deamidation percentage.

### Blood Experiments

In experiments involving blood, PDB_11_PEO_8_ (5 mg/mL w/v in pentane) was used to form the bilayer. Pores were allowed to insert in the presence of electrophysiology buffer (1 M NaCl, 50 mM TrisHCl pH 7.5). Upon insertion, an *I*/*V* curve and a blank recording were acquired. Then, the buffer in the *cis* compartment was partially replaced with defibrinated sheep blood and salt loss was compensated. For example, to obtain 25% (v/v) blood, 125 μL electrophysiology buffer was replaced with 100 μL defibrinated sheep blood and 25 μL of 4 M NaCl, 50 mM TrisHCl pH 7.5 (400 μL final volume, 1 M NaCl final concentration), and the solution was mixed well. All blood experiments were recorded using the sweep protocol (see Electrophysiology Data Recording and Analysis). Defibrinated sheep’s blood was used as the application of human blood is pending approval of the Ethics Committee.

### Data Processing and Machine Learning

#### Raw Trace Filtering

Raw electrophysiology traces were read from ABFv2 files by using the pyabf library. A processing pipeline was implemented by using the nanotrace library. First, the segment corresponding to the measurement potential (+30 mV) was extracted from each sweep. A low-pass Butterworth filter (implemented in SciPy) was then applied with a cutoff frequency of 10 kHz to remove high-frequency noise.

Next, the residual current [*I*_EX_ = ((*I*_O_ − *I*_B_)/*I*_O_) × 100%] was calculated by dividing the measured current (*I*_B_) by the baseline current (*I*_O_) to correct for pore-to-pore experimental variation. The baseline was calculated from the current between 60 and 120 pA after removing outliers using the median absolute deviation (MAD) criterion with a threshold value of 2.0.

After baseline determination for each sweep, the mean baseline was calculated by binning the baselines from all sweeps and selecting the highest bin. This step removes erroneously calculated baselines from sweeps that do not contain enough baseline samples. Finally, events were detected using a modified cumulative sum control chart (CUSUM) method, in which the control value is limited by a tunable ceiling value. The critical level parameter and ceiling value were tuned to 200 and 1000, respectively, with a baseline standard deviation of ∼0.5%. The low-pass pipeline stage was implemented using the scipy.signal.butter filter.

#### Feature Extraction

The feature set was defined a priori on the basis of the physical characteristics of nanopore blockade events rather than by automated feature selection. The 16 features comprise descriptors of blockade amplitude, event kinetics, waveform shape, and frequency-domain characteristics, and their relative contributions were subsequently evaluated by using permutation importance. Feature calculation was also included in the pipeline using the following functions: mean, median, std, ldt, _skew, kurt, clst_means, and psd_freq (i.e., *n* = 8 extracted feature parameters). _skew and kurtosis were implemented using scipy.stats.skew and scipy.stats.kurtosis, respectively. clst_means was implemented using the GaussianMixture model from scikit-learn. To account for different value ranges across feature spaces, all features were scaled independently to the same interquartile range and centered by removing the medians. Feature scaling was performed using RobustScaler from scikit-learn. Outlying events were removed from the feature matrix of each independent measurement using the EllipticEnvelope method for the reference insulin data, whereas the local outlier factor (scikit-learn) was used for the blood reference data because of its more complex distribution. Final training sets consisted of *n* = 1095 events from *N* = 5 pores for insulin and *n* = 14,547 events from *N* = 12 pores for blood-only samples.

#### Machine Learning

Distinguishing insulin from background events in 25% v/v blood was approached as an outlier-detection problem. Prior to training, events were filtered by an unsupervised outlier-removal step applied independently to each measurement (EllipticEnvelope for the insulin reference data and Local Outlier Factor for the blood reference data), removing atypical blockades such as gating, co-occupancy, and truncated events. First, reference insulin feature data were scaled to unit variance, and the EllipticEnvelope model from scikit-learn was fitted using a stratified *k*-fold split with *k* = 10 to avoid overfitting the training data. This effectively defined a radius in feature space within which insulin events were expected to fall. After the initial fit, the decision threshold was tuned to maximize the accuracy of distinguishing “insulin” from “blood” events. Accuracy was used as the metric to minimize false positives as much as possible during initial training. Decision-threshold tuning was performed using the minimize_scalar function from scipy.optimize and TunedThresholdClassifierCV from scikit-learn. Feature importance, i.e., the contribution of each feature to the classification performance of the model, was quantified using perturbation importance. In this approach, the values of each feature were shuffled one at a time while all others were kept fixed; the resulting reduction in classifier performance was used to quantify the importance of the corresponding feature. Permutation importance was calculated using permutation_importance from scikit-learn. The confusion matrix was calculated using confusion_matrix from scikit-learn. The learning curve was obtained by calculating the accuracy score on the reference insulin data as the training set and on the combined reference insulin and blood data as the test set.

#### Quantifying Insulin from Blood

Values for the calibration curve were calculated from the events predicted by the trained model to originate from the insulin molecules. This number was normalized to the total measurement time (minutes) for each independent experiment. Weighted linear regression was performed using scipy.optimize.curve_fit.

## Supplementary Material





## Notes

Full protein sequences, replicate measurements from electrophysiology experiments, voltage- and concentration-dependent protein capture data, mass spectrometry analyses (PDF)
1250223_insulin_IEX_protein_capture_matlab_script1_SAP V2.txt (TXT)
