# Supplementary material for: Engineered MspA Nanopores for Real-Time Detection of Insulin, Its Pharmaceutical Analogues, and Degradation Products
Source: ACS Nano. 2026 Oct 7;20(41):27841–54. doi: 10.1021/acsnano.6c10533 (PMC13689911; doi:10.1021/acsnano.6c10533)
Supplement: Supplementary file 1 [file nn6c10533_si_001.pdf]

Supporting Information

Engineered MspA Nanopores for Real-Time Detection of Insulin, Its Pharmaceutical Analogues, and Degradation Products

Sabine Straathof[1], Matthijs Tadema[1], Lijian Zhan[1] and Giovanni Maglia[1*]

[1] *Department of Chemical Biology, Groningen Biomolecular Sciences & Biotechnology Institute, University of Groningen, 9747 AG Groningen, The Netherlands*.

* Corresponding author. Email: giovanni.maglia@rug.nl

Supporting Table 1: Primary amino acid sequences of MspA mutants in this work. Mutations are in bold and underlined, in black up until M2, and in italic for additional mutations.

**MspA-D90N**<br>MGLDNELSLVDGQDRTLTVQQWDTFLNGVFPLDRNRLTREWFHSGRAKYIVAGPGADE<br>FEGTLELGYQIGFPWSLGVGINFSYTTPNILI**N**DGDITAPPFGLNSVITPNLFPGVSISADLG<br>NGPGIQEVATFSVDVSGAEGGVAVSNAHGTVTGAAGGVLLRPFARLIASTGDSVTTYGEP<br>WNMNGSAGSAWSHPQFEK*
**MspA-M2 (D90N-D91N-D93N-D118R-D134R-E139K)**<br>MGLDNELSLVDGQDRTLTVQQWDTFLNGVFPLDRNRLTREWFHSGRAKYIVAGPGADE<br>FEGTLELGYQIGFPWSLGVGINFSYTTPNILI**NN**G**N**ITAPPFGLNSVITPNLFPGVSISA**R**LG<br>NGPGIQEVATFSV**R**VSGA**K**GGVAVSNAHGTVTGAAGGVLLRPFARLIASTGDS<br>VTTYGEPWNMNGSAGSAWSHPQFEK*
**MspA-M2-S103K**<br>MGLDNELSLVDGQDRTLTVQQWDTFLNGVFPLDRNRLTREWFHSGRAKYIVAGPGADE<br>FEGTLELGYQIGFPWSLGVGINFSYTTPNILI**NN**G**N**ITAPPFGLN***K***VITPNLFPGVSISA**R**LG<br>NGPGIQEVATFSV**R**VSGA**K**GGVAVSNAHGTVTGAAGGVLLRPFARLIASTGDSVTTYGE<br>PWNMNGSAGSAWSHPQFEK*
**MspA-M2-I105K**<br>MGLDNELSLVDGQDRTLTVQQWDTFLNGVFPLDRNRLTREWFHSGRAKYIVAGPGADE<br>FEGTLELGYQIGFPWSLGVGINFSYTTPNILI**NN**G**N**ITAPPFGLNSV***K***TPNLFPGVSISA**R**L<br>GNGPGIQEVATFSV**R**VSGA**K**GGVAVSNAHGTVTGAAGGVLLRPFARLIASTGDSVTTYG<br>EPWNMNGSAGSAWSHPQFEK*
**MspA-M2-S103K-I105K**<br>MGLDNELSLVDGQDRTLTVQQWDTFLNGVFPLDRNRLTREWFHSGRAKYIVAGPGADE<br>FEGTLELGYQIGFPWSLGVGINFSYTTPNILI**NN**G**N**ITAPPFGLN***K***V***K***TPNLFPGVSISA**R**L<br>GNGPGIQEVATFSV**R**VSGA**K**GGVAVSNAHGTVTGAAGGVLLRPFARLIASTGDSVTTYG<br>EPWNMNGSAGSAWSHPQFEK*
**MspA-M2-S103K-N108K**<br>MGLDNELSLVDGQDRTLTVQQWDTFLNGVFPLDRNRLTREWFHSGRAKYIVAGPGADE<br>FEGTLELGYQIGFPWSLGVGINFSYTTPNILI**NN**G**N**ITAPPFGLN***K***VITP***K***LFPGVSISA**R**LG<br>NGPGIQEVATFSV**R**VSGA**K**GGVAVSNAHGTVTGAAGGVLLRPFARLIASTGDSVTTYGE<br>PWNMNGSAGSAWSHPQFEK*

Supporting Table 2: Insulin precursor and analogue characteristics.

Protein	Molecular weight (Da)	Charge at pH 7.5
Proinsulin human	9395	-3e
Insulin human	5808	-2e
Insulin aspart	5826	-3e
Insulin glargine	6051	0e

Supporting Table 3: Overview of insulin analogue separation by MspA mutants.
Abbreviations: insulin glargine (G), human insulin (H), insulin aspart (A).

Nanopore mutant	Reported P_{Cl-}/P_{K+}	Can separate G, H & A?	Required voltage for separation (mV)
MspA-M2-S103K-I105K	3.99 ± 0.09 (ref.[1])	Yes	20-50
MspA-M2-S103K-N108K	2.64 ± 0.07 (ref.[1])	Partial	100
MspA-M2-S103K	2.44 ± 0.06 (ref.[1])	Yes	80-120
MspA-M2-I105K	2.21 ± 0.03 (ref.[1])	Yes	40-80
MspA-M2	1.20 ± 0.01 (ref.[1])	Yes	200
MspA-D90N	~ 0.14 (ref.[2])	No	-

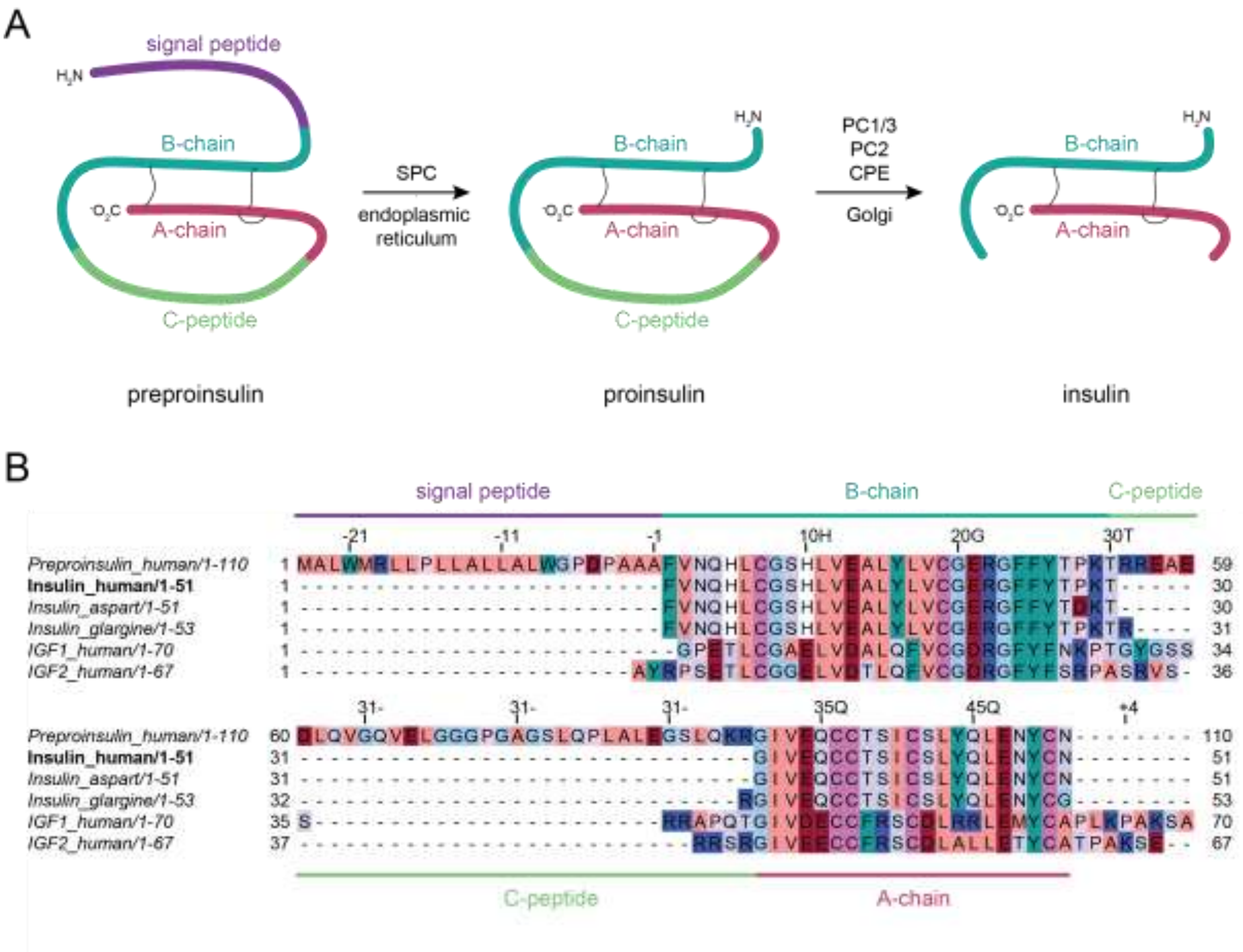

Supporting Figure 1: Insulin maturation and analogues. A) Insulin maturation. Signal peptide is in purple, A-chain is in pink, B-chain is in teal, C-peptide is in green. The signal peptide of preproinsulin is removed by the signal peptidase complex (SPC) in the endoplasmic reticulum to yield proinsulin.[3] Proinsulin is proteolytically cleaved in the Golgi by prohormone convertase 1/3 (PC1/3), prohormone convertase 2 (PC2), and carboxypeptidase E (CPE) to form mature insulin[4]. **B)** Primary sequence alignment of insulin precursors and analogues. Including preproinsulin (UniProt: P01308), insulin human, insulin aspart, insulin glargine, insulin-like growth factor 1 (IGF1, UniProt: P05019) and IGF2 (UniProt: P01344). Alignment was performed with Clustal Omega and image was generated with Jalview2a (ref.[5]).

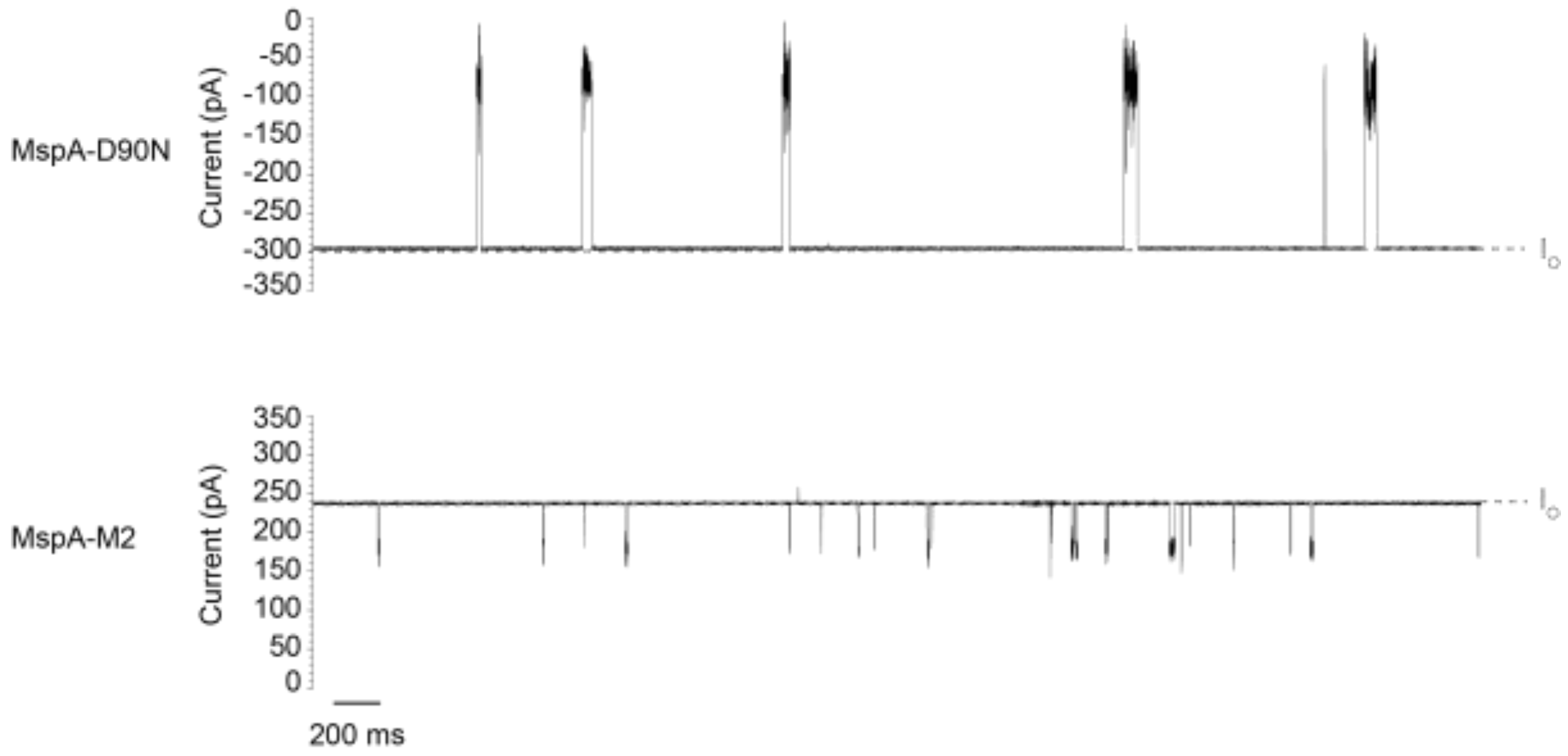

Supporting Figure 2: Electrophysiology traces of MspA-D90N and MspA-M2 capturing human insulin. Human insulin was added to *cis* at 250 nM. Measurements were conducted at -150 mV and +150 mV, respectively; in 1 M NaCl, 50 mM TrisHCl pH 7.5, with DPhPC composing the bilayer. Data were recorded at 50 kHz sampling rate, 10 kHz Bessel filter. Traces were additionally filtered with 2 kHz low-pass Gaussian filter for analysis and visualization.

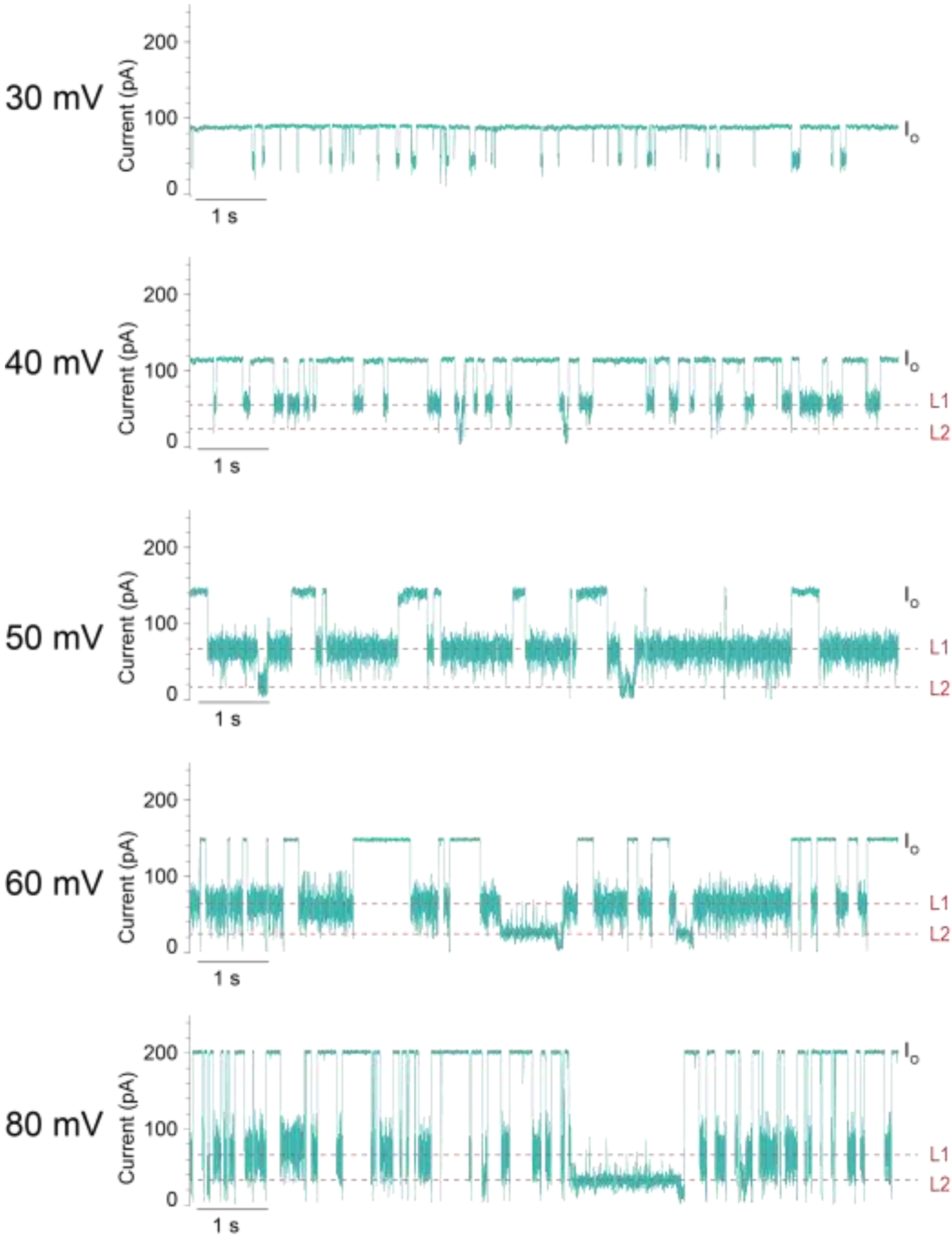

Supporting Figure 3: Electrophysiology traces of human insulin captured by MspA-M2-S103K-I105K at increasing voltage. I_O is indicated, level 1 and 2 (L1, L2) are indicated by red dotted line. Human insulin was added to *cis* at 250 nM. Measurements were conducted in 1 M NaCl, 50 mM TrisHCl pH 7.5, with DPhPC composing the bilayer. Data were recorded at 50 kHz sampling rate, 10 kHz Bessel filter. Traces were additionally filtered with 2 kHz low-pass Gaussian filter for visualization. Experiments were conducted at minimum in triplicate (N>3 pores).

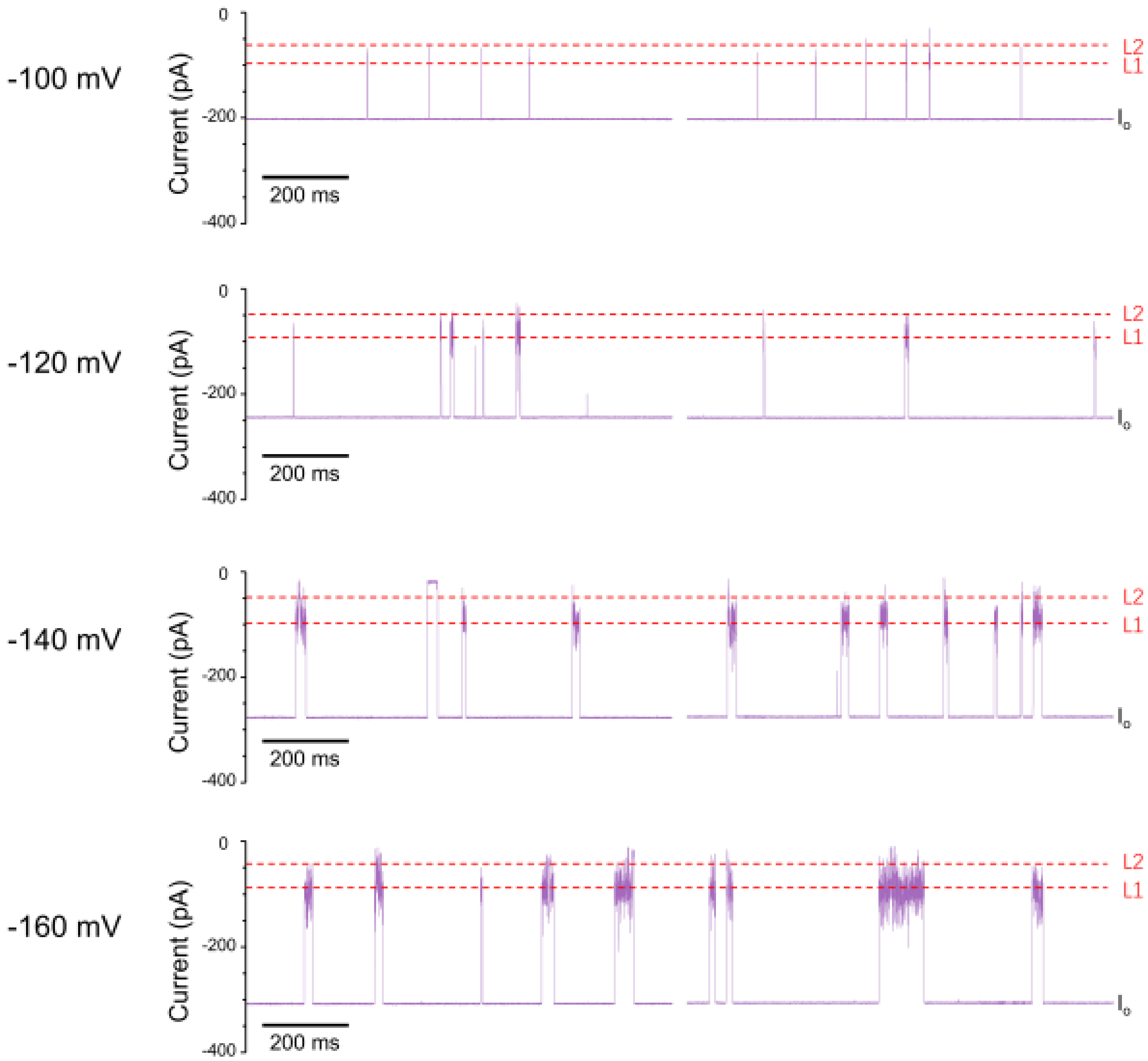

Supporting Figure 4: Electrophysiology traces of human insulin captured by MspA-D90N at increasing voltage. I_O is indicated, level 1 and 2 (L1, L2) are indicated by red dotted line. Human insulin was added to *cis* at 250 nM. Measurements were conducted in 1 M NaCl, 50 mM TrisHCl pH 7.5, with DPhPC composing the bilayer. Data were recorded at 50 kHz sampling rate, 10 kHz Bessel filter. Traces were additionally filtered with 2 kHz low-pass Gaussian filter for visualization. Experiments were conducted at minimum in triplicate (N>3 pores).

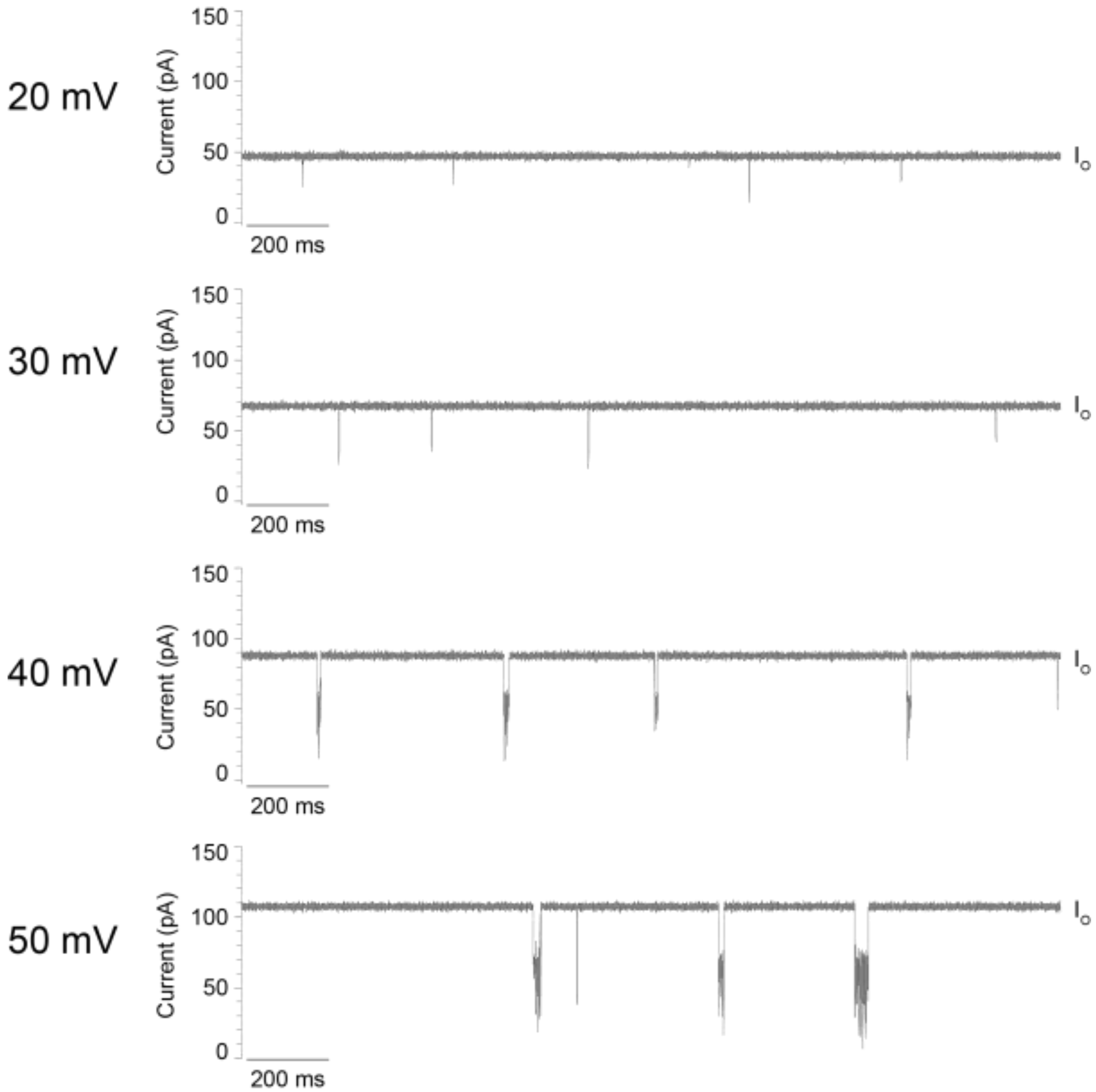

Supporting Figure 5: Electrophysiology traces of human insulin captured by MspA-M2-S103K-N108K at increasing voltage. I_O is indicated. Human insulin was added to *cis* at 250 nM. Measurements were conducted in 1 M NaCl, 50 mM TrisHCl pH 7.5, with DPhPC composing the bilayer. Data were recorded at 50 kHz sampling rate, 10 kHz Bessel filter. Traces were additionally filtered with 2 kHz low-pass Gaussian filter for visualization. Experiments were conducted at minimum in triplicate (N>3 pores).

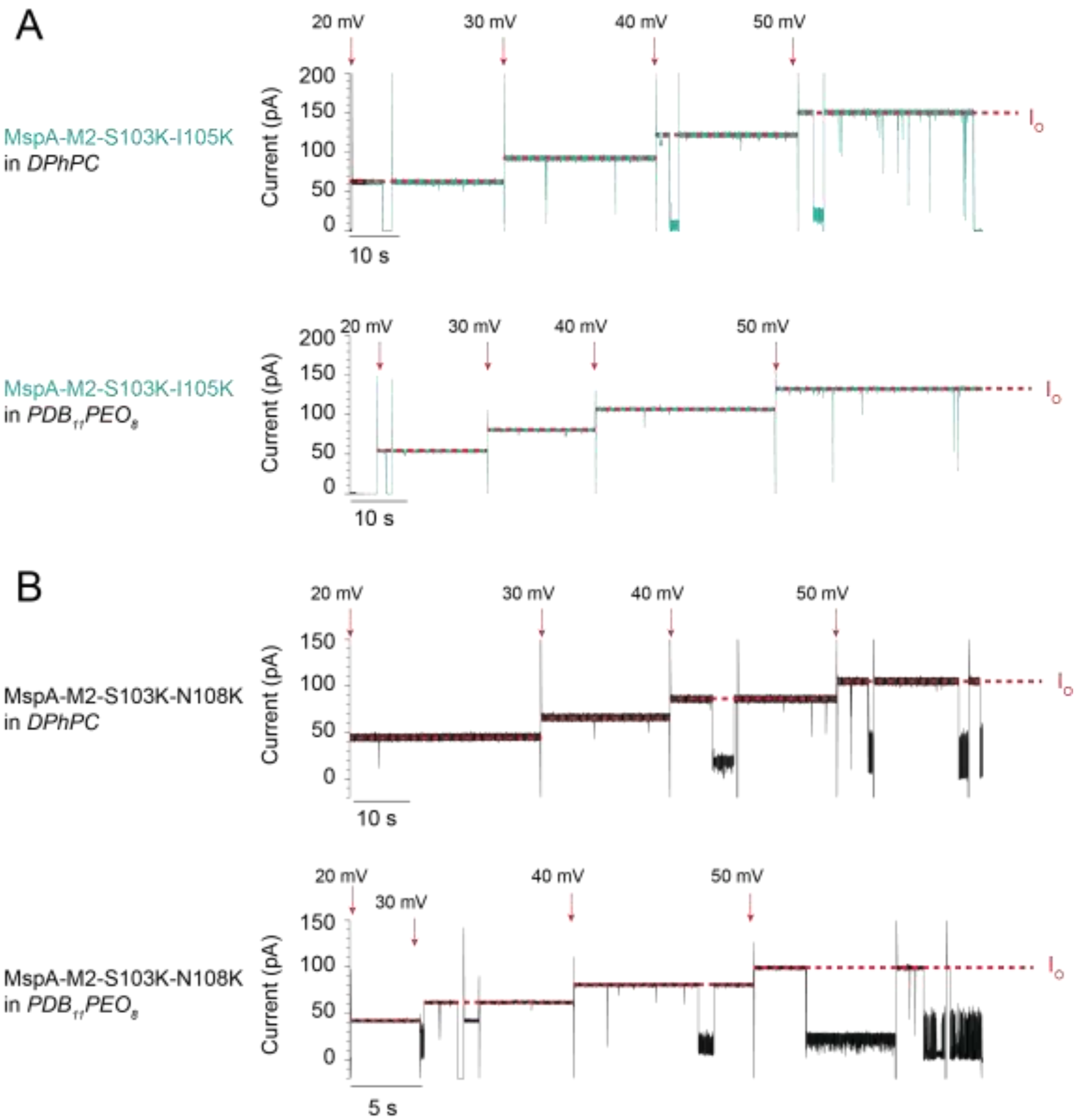

Supporting Figure 6: MspA-M2-double mutant in DPhPC and $PDB_{11}PEO_8$ bilayer. A) Electrophysiology blanks of MspA-M2-S103K-I105K in lipid (DPhPC) and polymer ($PDB_{11}PEO_8$) membranes. Externally applied bias is indicated. I_O is red dotted line. **B)** Electrophysiology blanks of MspA-M2-S103K-N108K in DPhPC and $PDB_{11}PEO_8$ membranes. Measurements were conducted in 1 M NaCl, 50 mM TrisHCl pH 7.5. Data were recorded at 50 kHz sampling rate, 10 kHz Bessel filter. Traces were additionally filtered with 2 kHz low-pass Gaussian filter for visualization. Experiments were conducted at minimum in triplicate (N>3 pores).

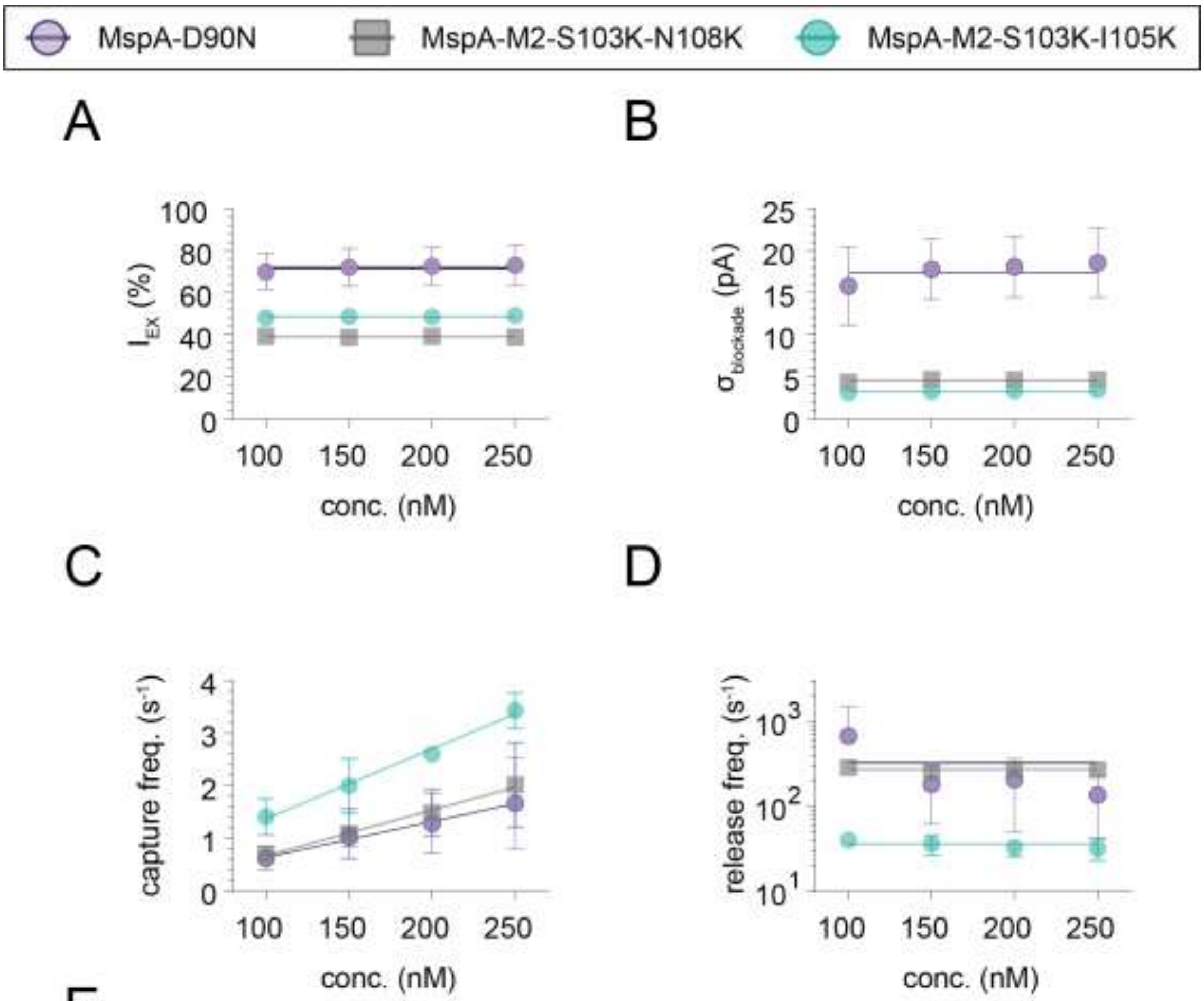

E

Concentration dependence		MspA-M2-S103K-I105K	MspA-M2-S103K-N108K	MspA-D90N
		μ ± SEM	μ ± SEM	μ ± SEM
A	I_{EX} (%)	48.5 ± 0.2	39.1 ± 0.1	71.7 ± 2.2
B	$\sigma_{blockade}$ (pA)	3.3 ± 0.1	4.6 ± 0.1	17.4 ± 1.0
C	k_{on} ($\mu M^{-1}.s^{-1}$)	13.3 ± 1.5	8.7 ± 2.4	6.8 ± 2.2
D	k_{off} (s^{-1})	35.9 ± 1.9	270.1 ± 4.8	325.4 ± 131.1

Supporting Figure 7: Concentration-dependent capture of human insulin by MspA mutants. A) Excluded current (I_{EX}), **B)** $\sigma_{blockade}$, **C)** capture frequency, **D)** release frequency of human insulin by respectively MspA-M2-S103K-I105K (teal) and MspA-M2-S103K-N108K (grey) and MspA-D90N (lilac) at increasing concentration. **E)** Overview of blockade parameters by the respective MspA M2 double mutants. The k_{on}, k_{off}, I_{EX} and $\sigma_{blockade}$ values (mean μ ± standard error of the mean SEM) were obtained by least-squared linear regression analysis in GraphPad 9.5.0. Human insulin was added to *cis*. Measurements were conducted at 30 mV, in 1 M NaCl, 50 mM TrisHCl pH 7.5. Data were recorded at 50 kHz sampling rate, 10 kHz Bessel filter. Traces were additionally filtered with 2 kHz low-pass Gaussian filter for analysis and visualization. Measurements were conducted at +30 mV (MspA-M2 mutants) and −120 mV (MspA-D90N). Experiments were performed at minimum in triplicate (N ≥ 3 pores), with n > 500 events per data point for MspA-M2-S103K-I105K (teal) and MspA-M2-S103K-N108K, and n > 112 events per data point for MspA-D90N. Error bars in A-D represent standard deviation, and in E standard error of the mean.

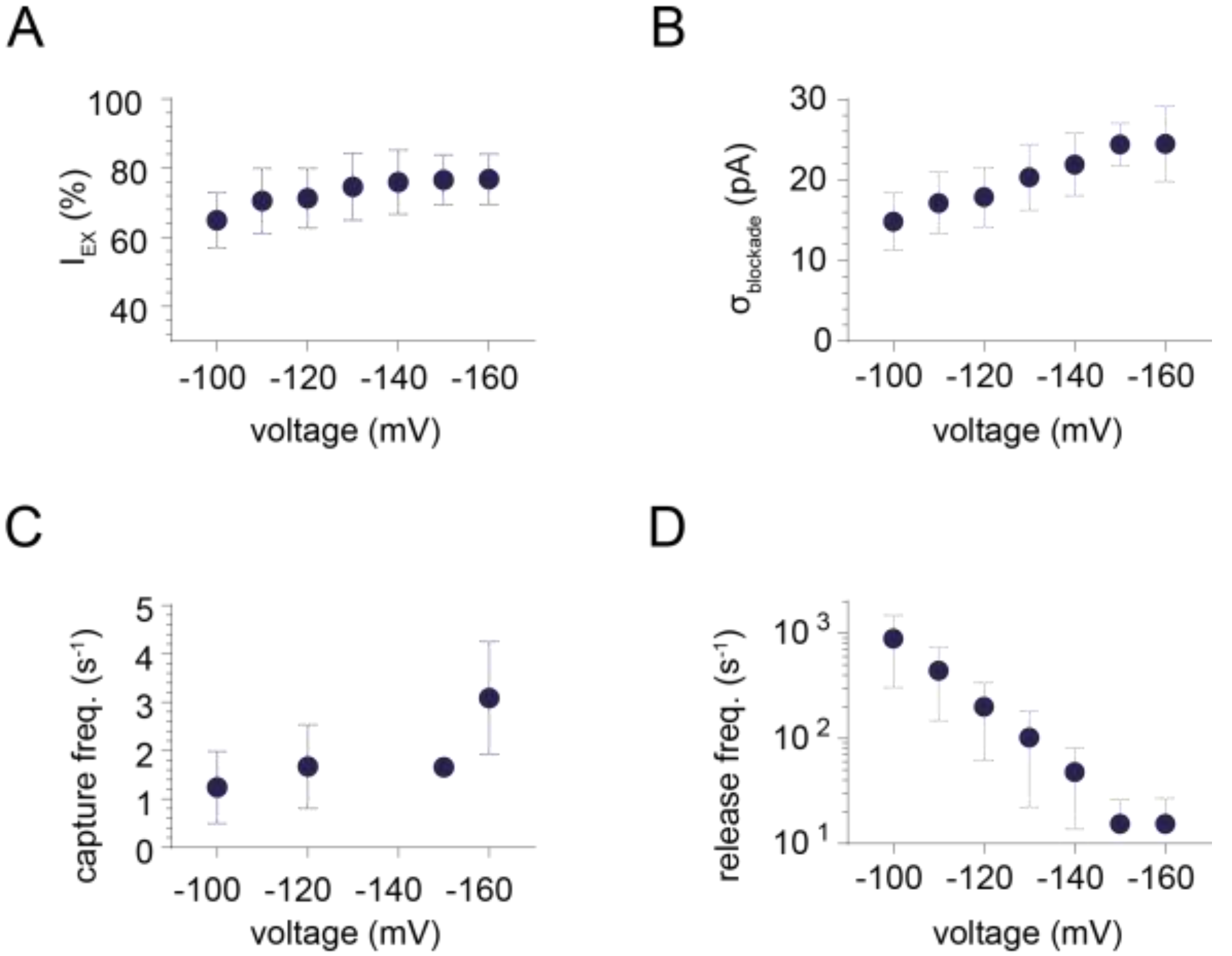

Supporting Figure 8: Voltage-dependent capture of human insulin by MspA-D90N. **A)** I_{EX}, **B)** $\sigma_{blockade}$, **C)** capture frequency, **D)** release frequency of human insulin at increasing voltage. Human insulin was added to *cis* at 250 nM. Measurements were conducted in 1 M NaCl, 50 mM TrisHCl pH 7.5, with DPhPC composing the bilayer. Data were recorded at 50 kHz sampling rate, 10 kHz Bessel filter. Traces were additionally filtered with 2 kHz low-pass Gaussian filter for analysis and visualization. Experiments were conducted in triplicate, with n >133 events per data point. Error bars represent standard deviation.

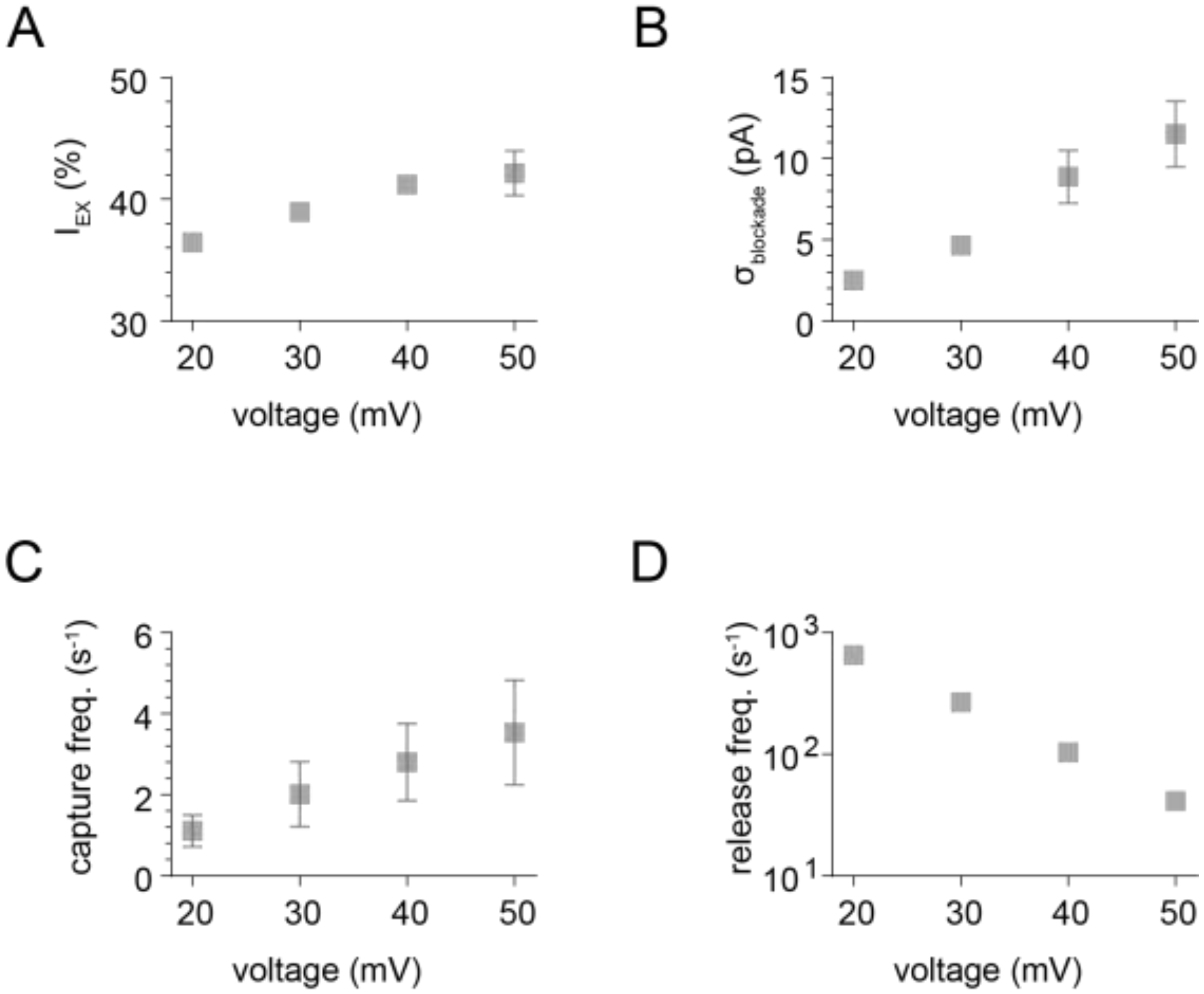

Supporting Figure 9: Voltage-dependent capture of human insulin by MspA-M2-S103K-N108K. A) I_{EX}, **B)** $\sigma_{blockade}$, **C)** capture frequency, **D)** release frequency of human insulin at increasing voltage. Human insulin was added to *cis* at 250 nM. Measurements were conducted in 1 M NaCl, 50 mM TrisHCl pH 7.5. Data were recorded at 50 kHz sampling rate, 10 kHz Bessel filter. Traces were additionally filtered with 2 kHz low-pass Gaussian filter for analysis and visualization. Experiments were conducted at minimum in triplicate (N>3 pores). Error bars represent standard deviation.

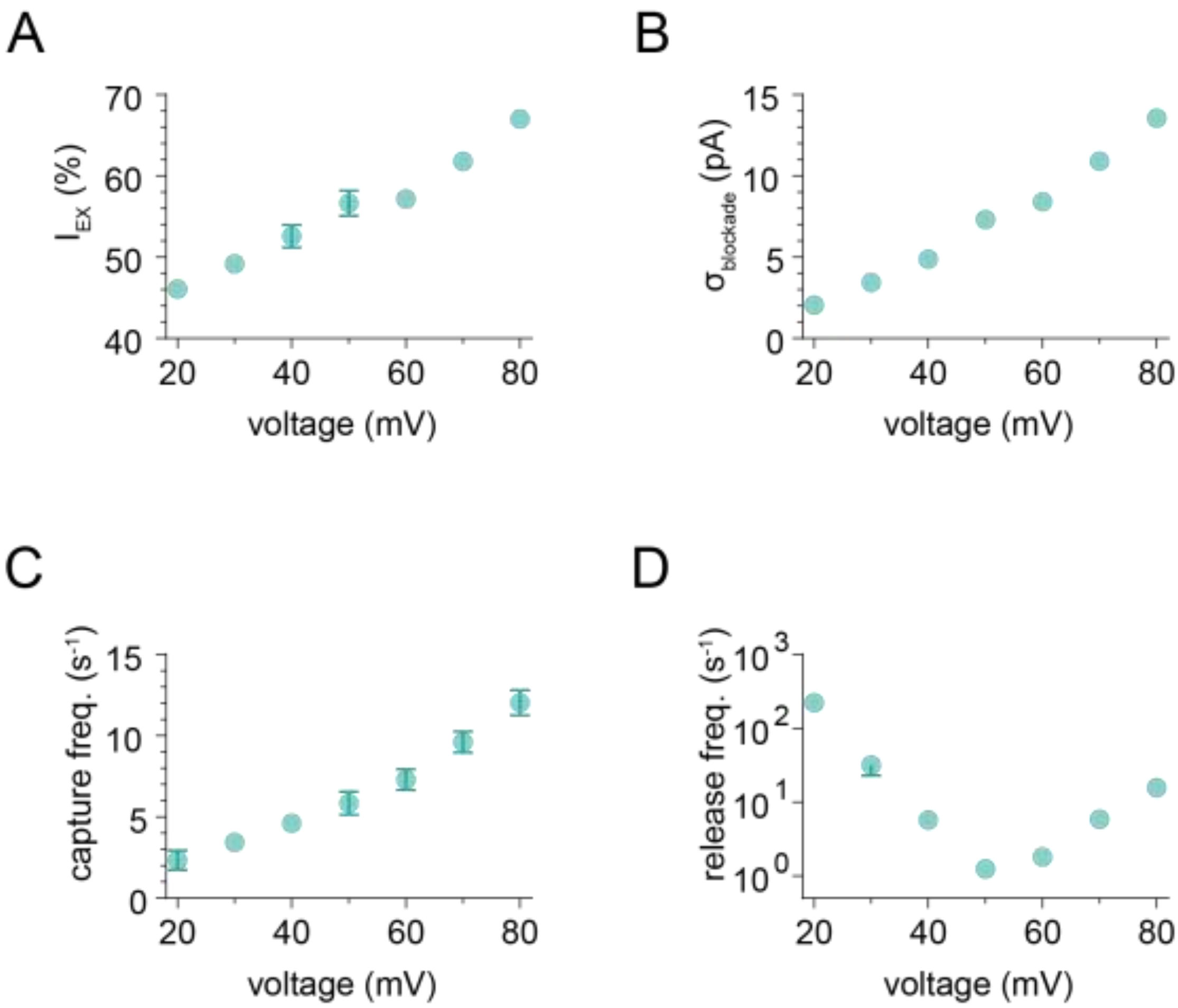

Supporting Figure 10: Voltage-dependent capture of human insulin by MspA-M2-S103K-I105K. A) I_{EX}, **B)** $\sigma_{blockade}$, **C)** capture frequency, **D)** release frequency of human insulin at increasing voltage. Human insulin was added to *cis* at 250 nM. Measurements were conducted in 1 M NaCl, 50 mM TrisHCl pH 7.5. Data were recorded at 50 kHz sampling rate, 10 kHz Bessel filter. Traces were additionally filtered with 2 kHz low-pass Gaussian filter for analysis and visualization. Experiments were conducted at minimum in triplicate (N>3 pores). Error bars represent standard deviation.

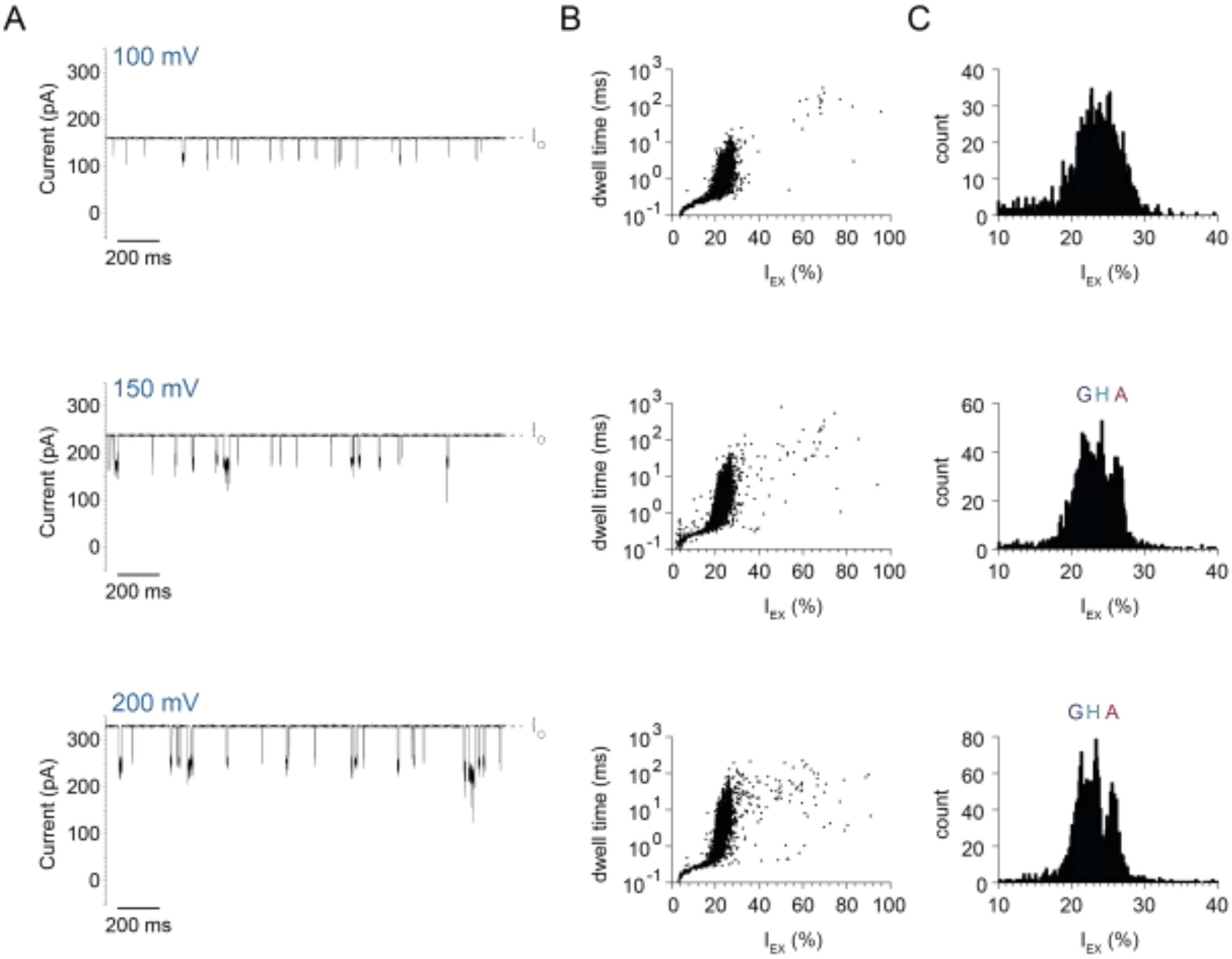

Supporting Figure 11: Insulin analogue separation by MspA-M2. Reported selectivity P_{Cl-}/P_{K+} = 1.20 (ref.[1]). **A)** Electrophysiology trace of MspA-M2 capturing a mix of human insulin, insulin aspart and insulin glargine. Externally applied voltage is indicated in blue. I_O is grey dotted line. **B)** Corresponding scatterplot with n>500 datapoints. **C)** Corresponding I_{EX}-histogram. Peaks are annotated with insulin glargine (G, dark blue), human insulin (H, teal), and insulin aspart (A, burgundy). Equal molar mixture of human insulin, insulin aspart, insulin glargine were added to *cis*, at 250 nM each. Measurements were performed at a externally applied voltage where there was sufficient capture and release of the proteins. Measurements were conducted in 1 M NaCl, 50 mM TrisHCl pH 7.5. Data were recorded at 50 kHz sampling rate, 10 kHz Bessel filter. Traces were additionally filtered with 2 kHz low-pass Gaussian filter for visualization and analysis.

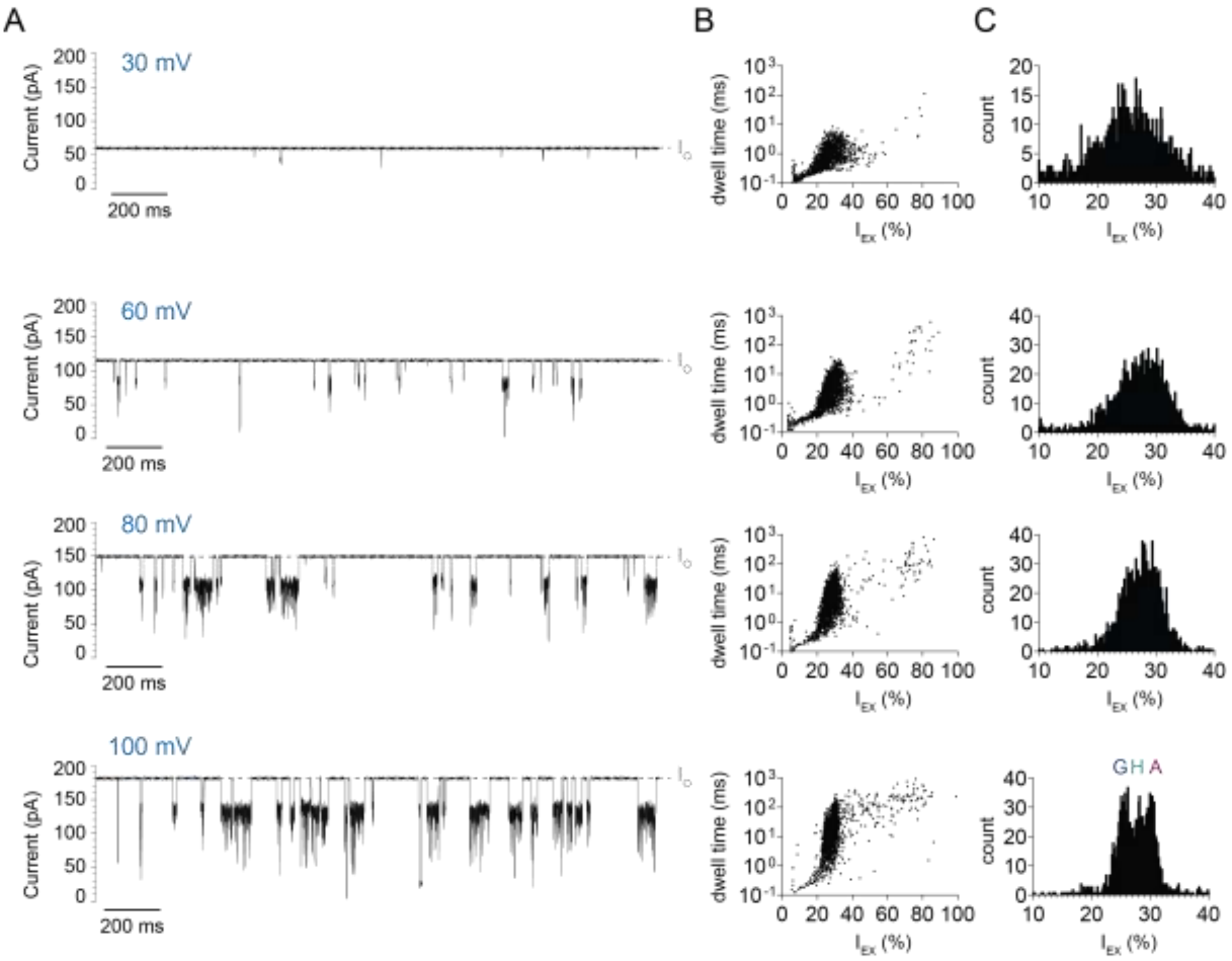

Supporting Figure 12: Insulin analogue separation by MspA-M2-S103K-N108K. Reported selectivity P_{Cl-}/P_{K+} = 2.64 (ref.[1]). **A)** Electrophysiology trace of MspA-M2-S103K-N108K capturing a mix of human insulin, insulin aspart and insulin glargine. Externally applied voltage is indicated in blue. I_O is grey dotted line. **B)** Corresponding scatterplot with n>500 datapoints. **C)** Corresponding I_{EX}-histogram. Peaks are annotated with insulin glargine (G, dark blue), human insulin (H, teal), and insulin aspart (A, burgundy). Equal molar mixture of human insulin, insulin aspart, insulin glargine were added to *cis*, at 250 nM each. Measurements were performed at a externally applied voltage where there was sufficient capture and release of the proteins. Measurements were conducted in 1 M NaCl, 50 mM TrisHCl pH 7.5. Data were recorded at 50 kHz sampling rate, 10 kHz Bessel filter. Traces were additionally filtered with 2 kHz low-pass Gaussian filter for visualization and analysis.

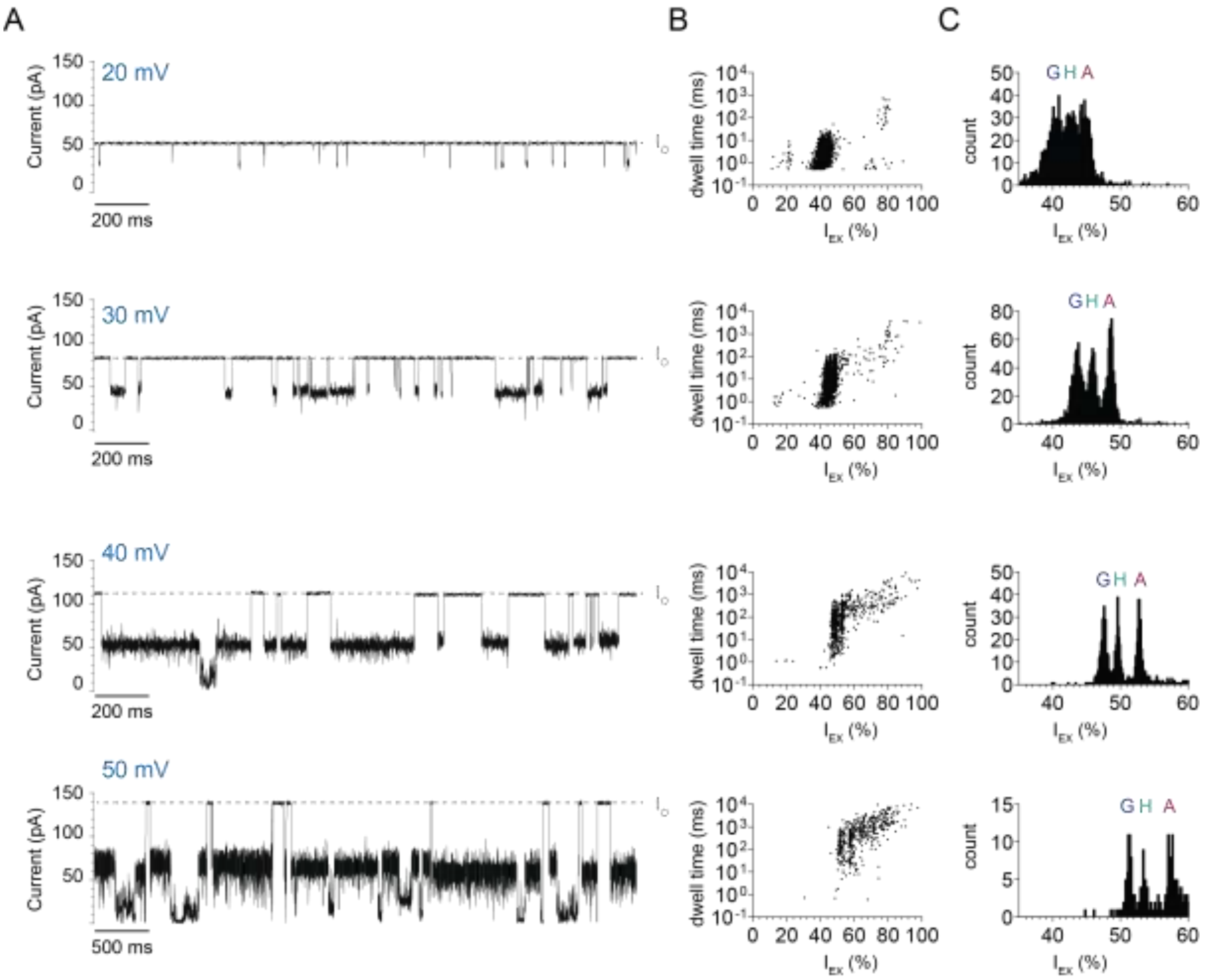

Supporting Figure 13: Insulin analogue separation by MspA-M2-S103K-I105K. Reported selectivity P_{Cl-}/P_{K+} = 3.99 (ref.[1]). **A)** Electrophysiology trace of MspA-M2-S103K-I105K capturing a mix of human insulin, insulin aspart and insulin glargine. Externally applied voltage is indicated in blue. I_O is grey dotted line. **B)** Corresponding scatterplot with n>500 datapoints. **C)** Corresponding I_{EX}-histogram. Peaks are annotated with insulin glargine (G, dark blue), human insulin (H, teal), and insulin aspart (A, burgundy). Equal molar mixture of human insulin, insulin aspart, insulin glargine were added to *cis*, at 250 nM each. Measurements were performed at a externally applied voltage where there was sufficient capture and release of the proteins. Measurements were conducted in 1 M NaCl, 50 mM TrisHCl pH 7.5. Data were recorded at 50 kHz sampling rate, 10 kHz Bessel filter. Traces were additionally filtered with 2 kHz low-pass Gaussian filter for visualization and analysis.

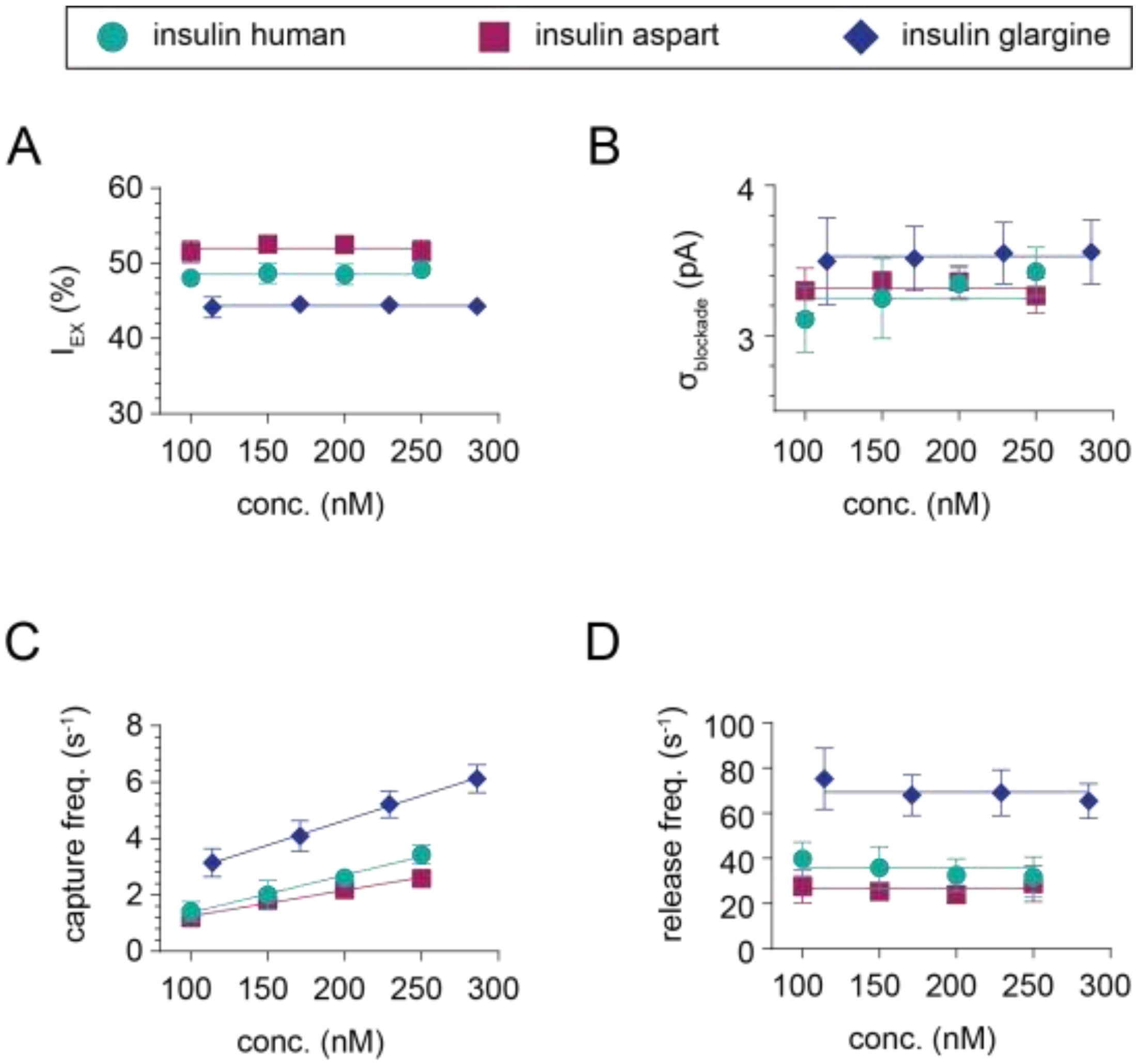

E

Concentration dependence		Human (-2e)	Aspart (-3e)	Glargine (0e)
		μ ± SEM	μ ± SEM	μ ± SEM
A	I_{EX} (%)	48.5 ± 0.2	52.0 ± 0.3	44.4 ± 0.2
B	$\sigma_{blockade}$ (pA)	3.3 ± 0.1	3.3 ± 0.0	3.5 ± 0.1
C	k_{on} ($\mu M^{-1}.s^{-1}$)	13.3 ± 1.5	9.2 ± 0.7	17.5 ± 2.0
D	k_{off} (s^{-1})	35.9 ± 1.9	26.7 ± 1.5	69.4 ± 2.8

Supporting Figure 14: Concentration-dependent capture of insulin analogues by MspA-M2-S103K-I105K$^{2.9*}$. A) I_{EX}, **B)** $\sigma_{blockade}$, **C)** capture frequency, **D)** release frequency of respectively insulin human (teal), insulin aspart (burgundy), and glargine (dark blue) by MspA-M2-S103K-I105K at increasing concentration. **E)** Overview of blockade parameters by the respective insulin analogues. The k_{on}, k_{off}, I_{EX} and $\sigma_{blockade}$ values (mean μ ± standard error of the mean SEM) were obtained by least-squared linear regression analysis in GraphPad 9.5.0. Insulin analogues were added separately to *cis*. Measurements were conducted at 30 mV, in 1 M NaCl, 50 mM TrisHCl pH 7.5. Data were recorded at 50 kHz sampling rate, 10 kHz Bessel filter. Traces were additionally filtered with 2 kHz low-pass Gaussian filter for analysis and visualization. Experiments were conducted at minimum in triplicate (N>3 pores). Error bars in A-D represent standard deviation, and in E standard error of the mean.

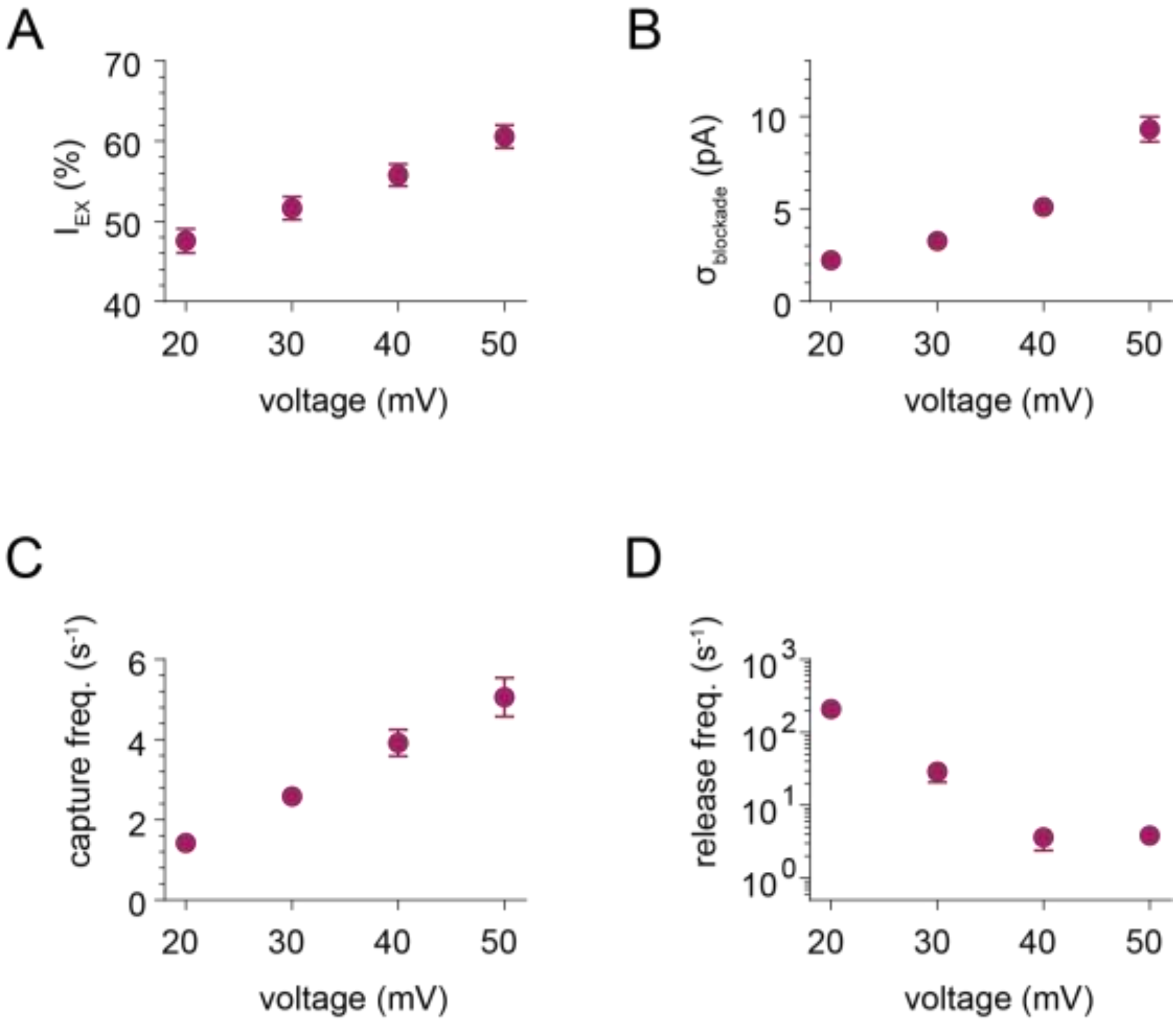

Supporting Figure 15: Voltage-dependent capture of insulin aspart by MspA-M2-S103K-I105K. A) I_{EX}, **B)** $\sigma_{blockade}$, **C)** capture frequency, **D)** release frequency of insulin aspart at increasing voltage. Insulin aspart was added to *cis* at 250 nM. Measurements were conducted in 1 M NaCl, 50 mM TrisHCl pH 7.5. Data were recorded at 50 kHz sampling rate, 10 kHz Bessel filter. Traces were additionally filtered with 2 kHz low-pass Gaussian filter for analysis and visualization. Experiments were conducted at minimum in triplicate (N>3 pores). Error bars represent standard deviation.

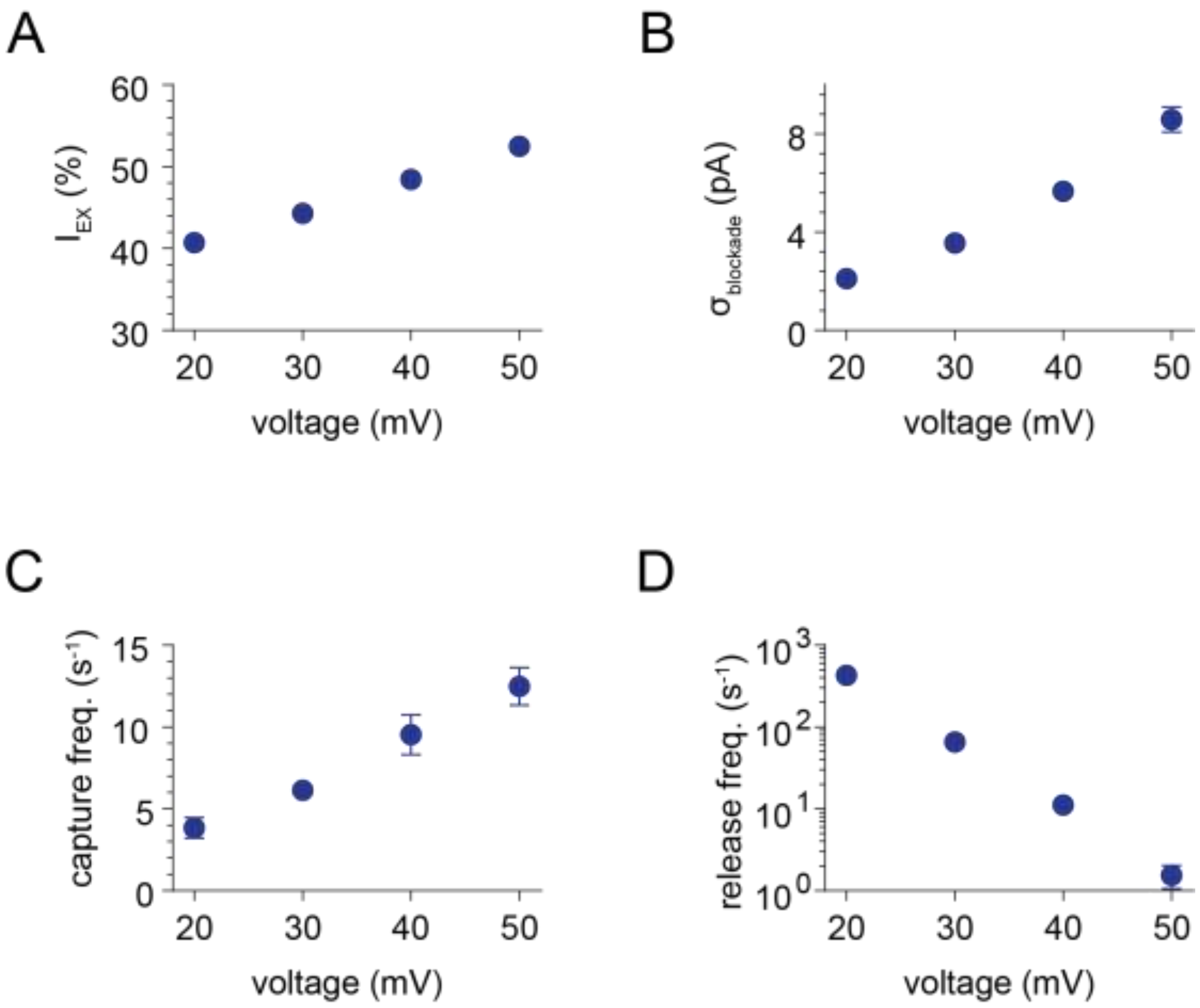

Supporting Figure 16: Voltage-dependent capture of insulin glargine by MspA-M2-S103K-I105K. A) I_{EX}, **B)** $\sigma_{blockade}$, **C)** capture frequency, **D)** release frequency of insulin glargine at increasing voltage. Insulin glargine was added to *cis* at 286 nM. Measurements were conducted in 1 M NaCl, 50 mM TrisHCl pH 7.5, with $PDB_{11}PEO_8$ composing the bilayer. Data were recorded at 50 kHz sampling rate, 10 kHz Bessel filter. Traces were additionally filtered with 2 kHz low-pass Gaussian filter for analysis and visualization. Experiments were conducted at minimum in triplicate (N>3 pores). Error bars represent standard deviation.

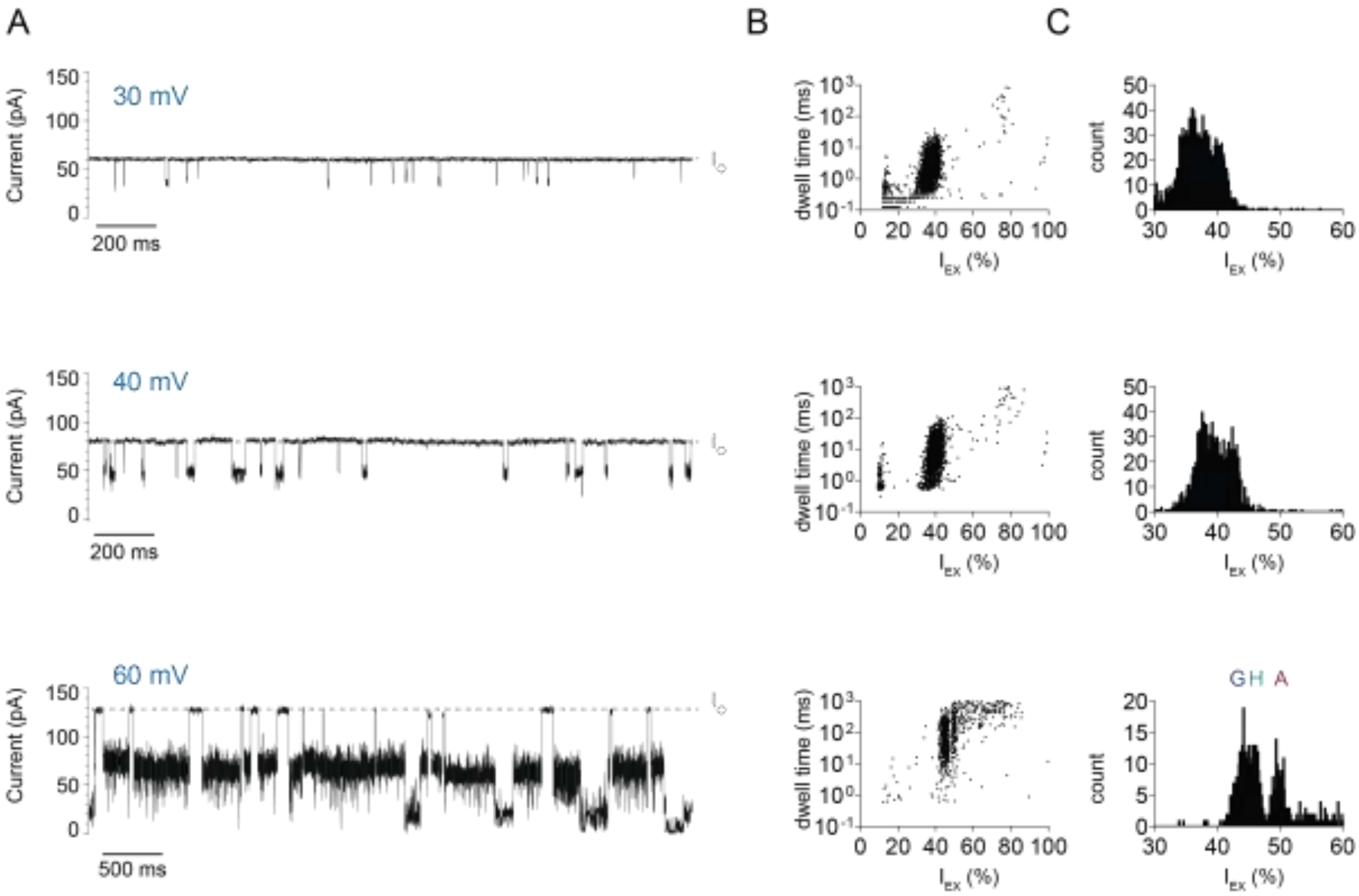

Supporting Figure 17: Insulin analogue separation by MspA-M2-I105K. Reported selectivity P_{Cl-}/P_{K+} = 2.21 (ref.[1]). **A)** Electrophysiology trace of MspA-M2-I105K capturing a mix of human insulin, insulin aspart and insulin glargine. Externally applied voltage is indicated in blue. I_O is grey dotted line. **B)** Corresponding scatterplot with n>500 datapoints. **C)** Corresponding I_{EX}-histogram. Peaks are annotated with insulin glargine (G, dark blue), human insulin (H, teal), and insulin aspart (A, burgundy). Equal molar mixture of human insulin, insulin aspart, insulin glargine were added to *cis*, at 250 nM each. Measurements were performed at a externally applied voltage where there was sufficient capture and release of the proteins. Measurements were conducted in 1 M NaCl, 50 mM TrisHCl pH 7.5. Data were recorded at 50 kHz sampling rate, 10 kHz Bessel filter. Traces were additionally filtered with 2 kHz low-pass Gaussian filter for visualization and analysis.

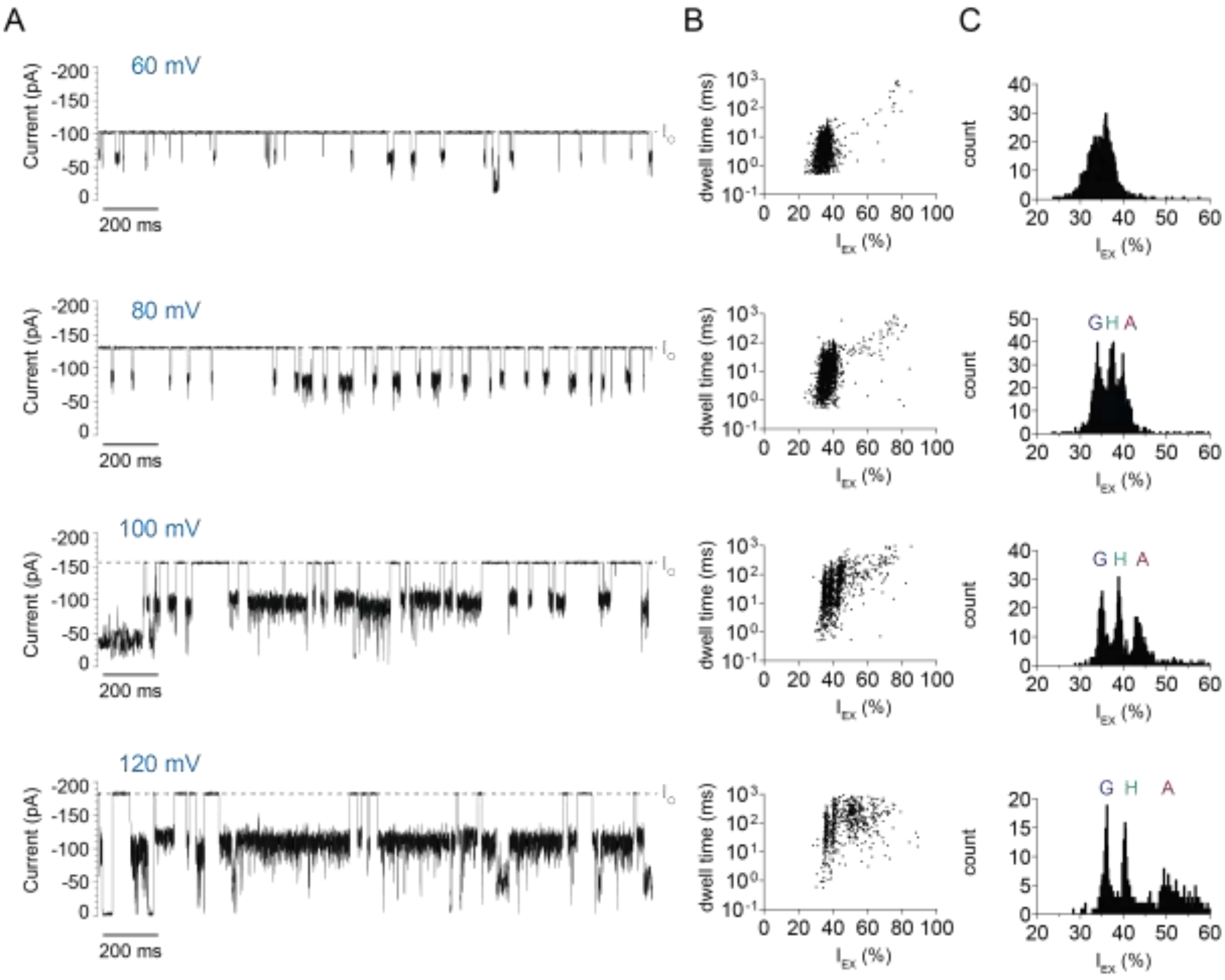

Supporting Figure 18: Insulin analogue separation by MspA-M2-S103K. Reported selectivity P_{Cl-}/P_{K+} = 2.44 (ref.[1]). **A)** Electrophysiology trace of MspA-M2-S103K capturing a mix of human insulin, insulin aspart and insulin glargine. Externally applied voltage is indicated in blue. I_O is grey dotted line. **B)** Corresponding scatterplot with n>500 datapoints. **C)** Corresponding I_{EX}-histogram. Peaks are annotated with insulin glargine (G, dark blue), human insulin (H, teal), and insulin aspart (A, burgundy). Equal molar mixture of human insulin, insulin aspart, insulin glargine were added to *cis*, at 250 nM each. Measurements were performed at a externally applied voltage where there was sufficient capture and release of the proteins. Measurements were conducted in 1 M NaCl, 50 mM TrisHCl pH 7.5. Data were recorded at 50 kHz sampling rate, 10 kHz Bessel filter. Traces were additionally filtered with 2 kHz low-pass Gaussian filter for visualization and analysis.

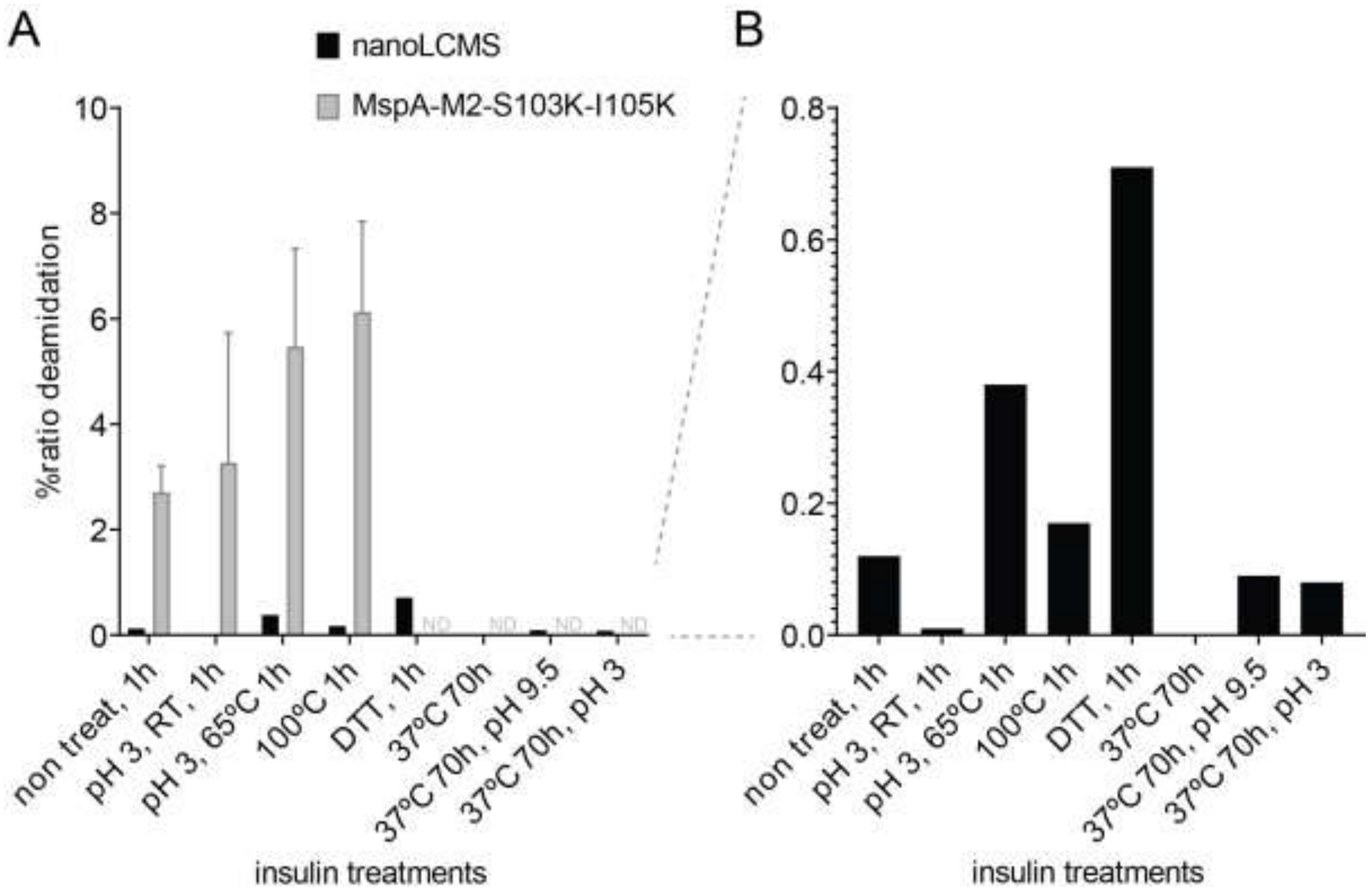

Supporting Figure 19: Insulin deamidation events detected by nano-LC-MS compared to detection by MspA-M2-S103K-I105K. A) %ratio of deamidated insulin from various protein-treatments (see Methods), as detected by nano-LC-MS (black) and by MspA-M2-S103K-I105K (grey). ND = not determined. B) zoom-in of %ratio of deamidated insulin as measured by nano-LC-MS.

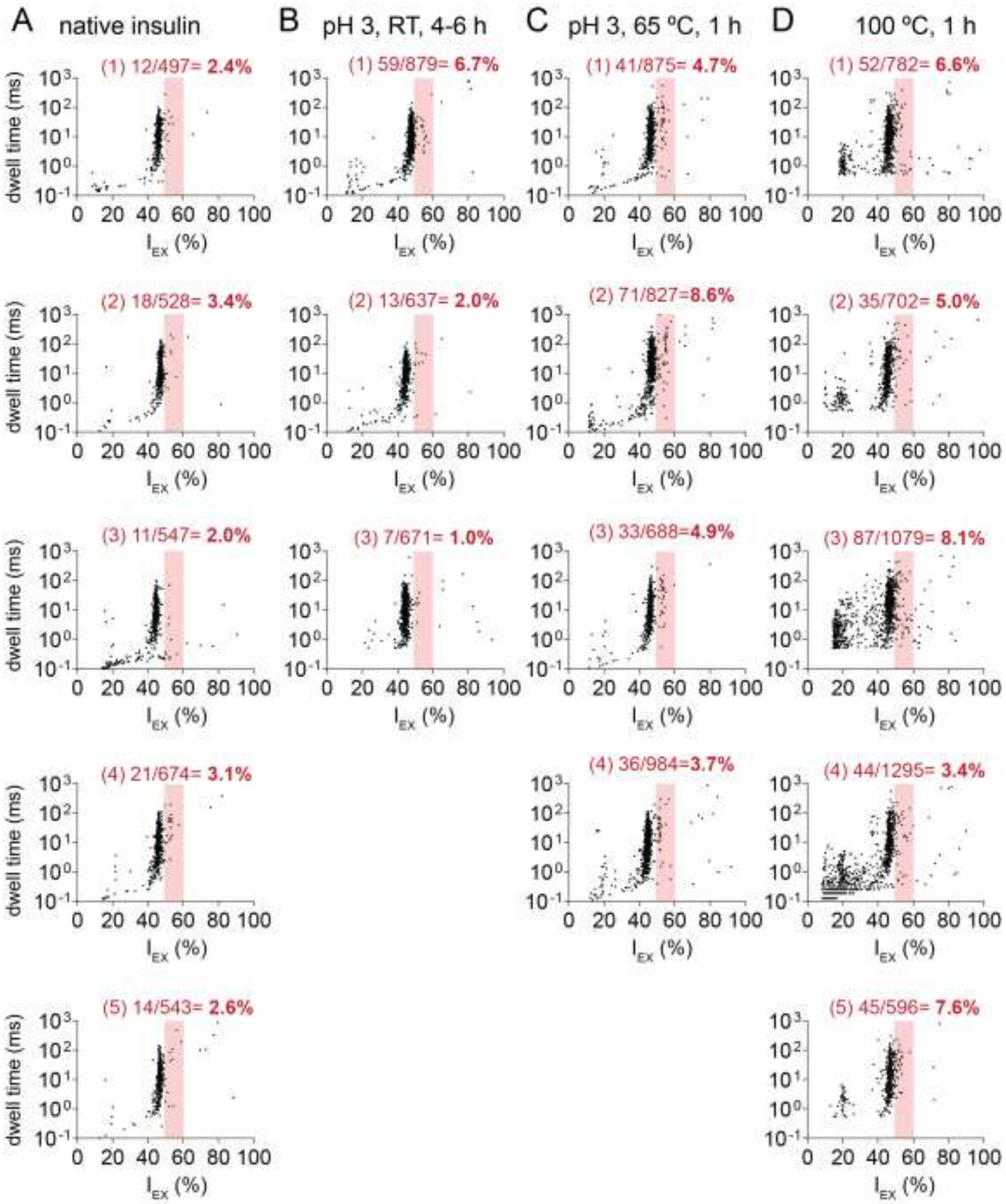

Supporting Figure 20: Insulin deamidation detection by MspA-M2-S103K-I105K[2.6*]. Extended data from Figure 4B–E. Scatterplot of separate measurements of **A)** native, untreated insulin, **B)** insulin pre-incubated at low pH (50 mM acetic acid pH 3.0, 4-6 h at room temperature), **C)** insulin pre-incubated in low pH and high temperature (50 mM acetic acid pH 3.0, 1 h at 65 ºC), **D)** insulin pre-incubated at 100 ºC (1 h). Red block indicates expected deaminated insulin blockades (I_{EX} 48.934-60%), and red text indicates the ratio (%) of "deaminated" events over the total events, per replicate. Insulin (10 µM) was pre-incubated as stated, and then added to *cis* at 250 nM. Measurements were conducted at 30 mV in 1 M NaCl, 50 mM TrisHCl pH 7.5, with $PDB_{11}PEO_8$ composing the bilayer. Data were recorded at 50 kHz sampling rate, 10 kHz Bessel filter. Traces were additionally filtered with 2 kHz low-pass Gaussian filter for visualization and analysis. Experiments were executed at least in triplicate (N>3 pores).

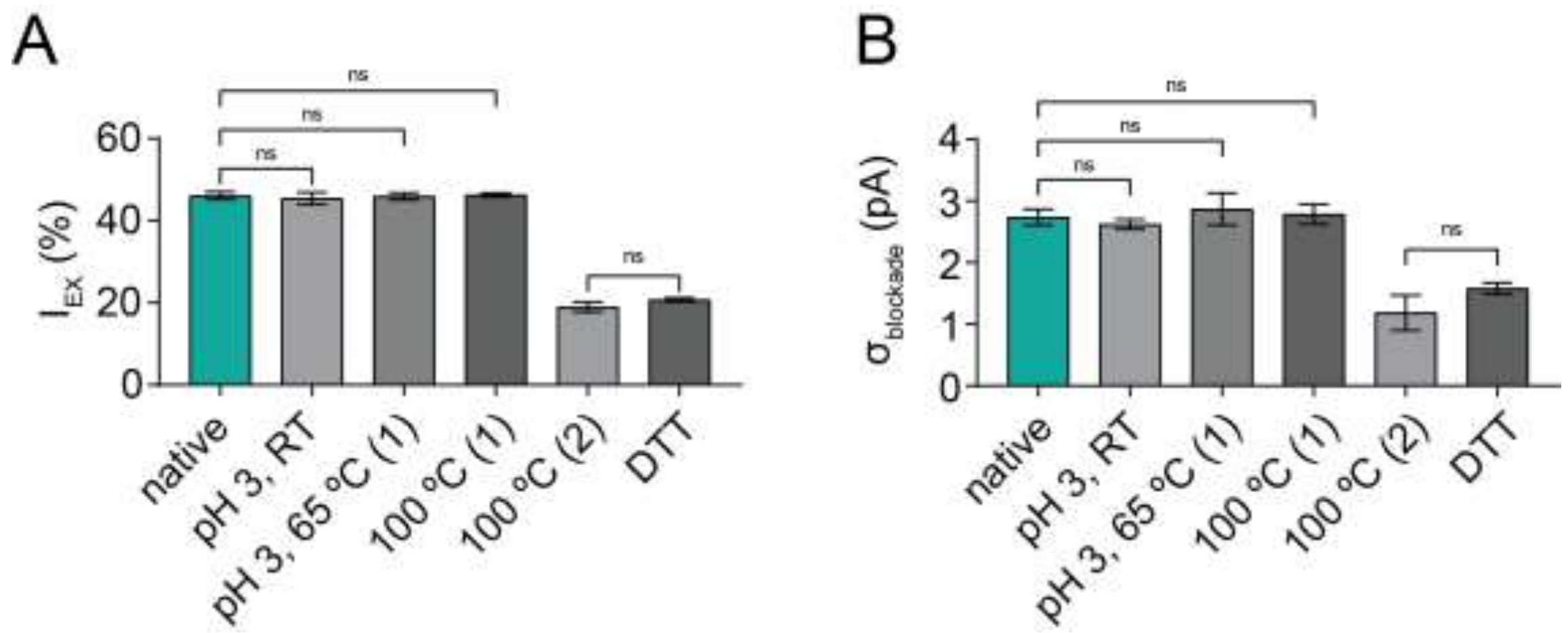

Supporting Figure 21: Insulin treatment bulk comparison. A) I_{EX} and **B)** $\sigma_{blockade}$ of native human insulin (teal) and pre-incubated human insulin (greys) electrophysiology blockades. Error bars indicate standard deviation. Unpaired t-test indicated non-significant (ns) difference between the groups. Insulin (10 μM) was pre-incubated as indicated, and then added to *cis* at 250 nM. Measurements were conducted at 30 mV in 1 M NaCl, 50 mM TrisHCl pH 7.5, with $PDB_{11}PEO_8$ composing the bilayer. Data were recorded at 50 kHz sampling rate, 10 kHz Bessel filter. Traces were additionally filtered with 2 kHz low-pass Gaussian filter for visualization and analysis. Experiments were executed at least in triplicate (N>3 pores).

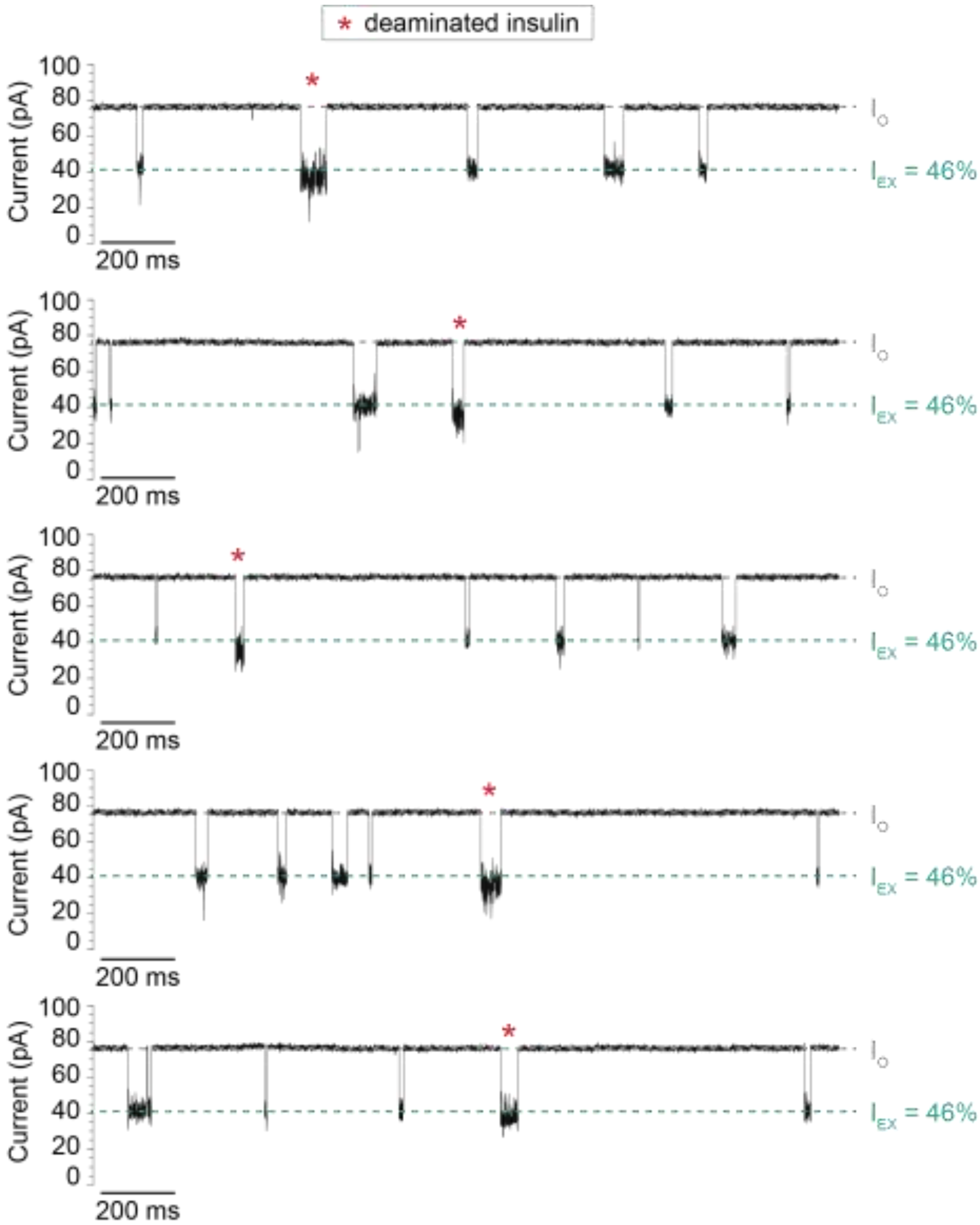

Supporting Figure 22: Insulin deamidation events by MspA-M2-S103K-I105K[2.6*].
Electrophysiology events of human insulin pre-incubated in low pH and high temperature (50 mM acetic acid pH 3.0, 1 h at 65 ºC). Red asterisk indicates deamidation events (I_{EX} 48.934-60%). I_O is indicated by grey dotted line. I_{EX} at 46% of native insulin is in teal. Insulin (10 µM) was pre-incubated as stated, and then added to *cis* at 250 nM. Measurements were conducted at 30 mV in 1 M NaCl, 50 mM TrisHCl pH 7.5, with $PDB_{11}PEO_8$ composing the bilayer. Data were recorded at 50 kHz sampling rate, 10 kHz Bessel filter. Traces were additionally filtered with 2 kHz low-pass Gaussian filter for visualization and analysis. Experiments were executed at least in triplicate (N>3 pores).

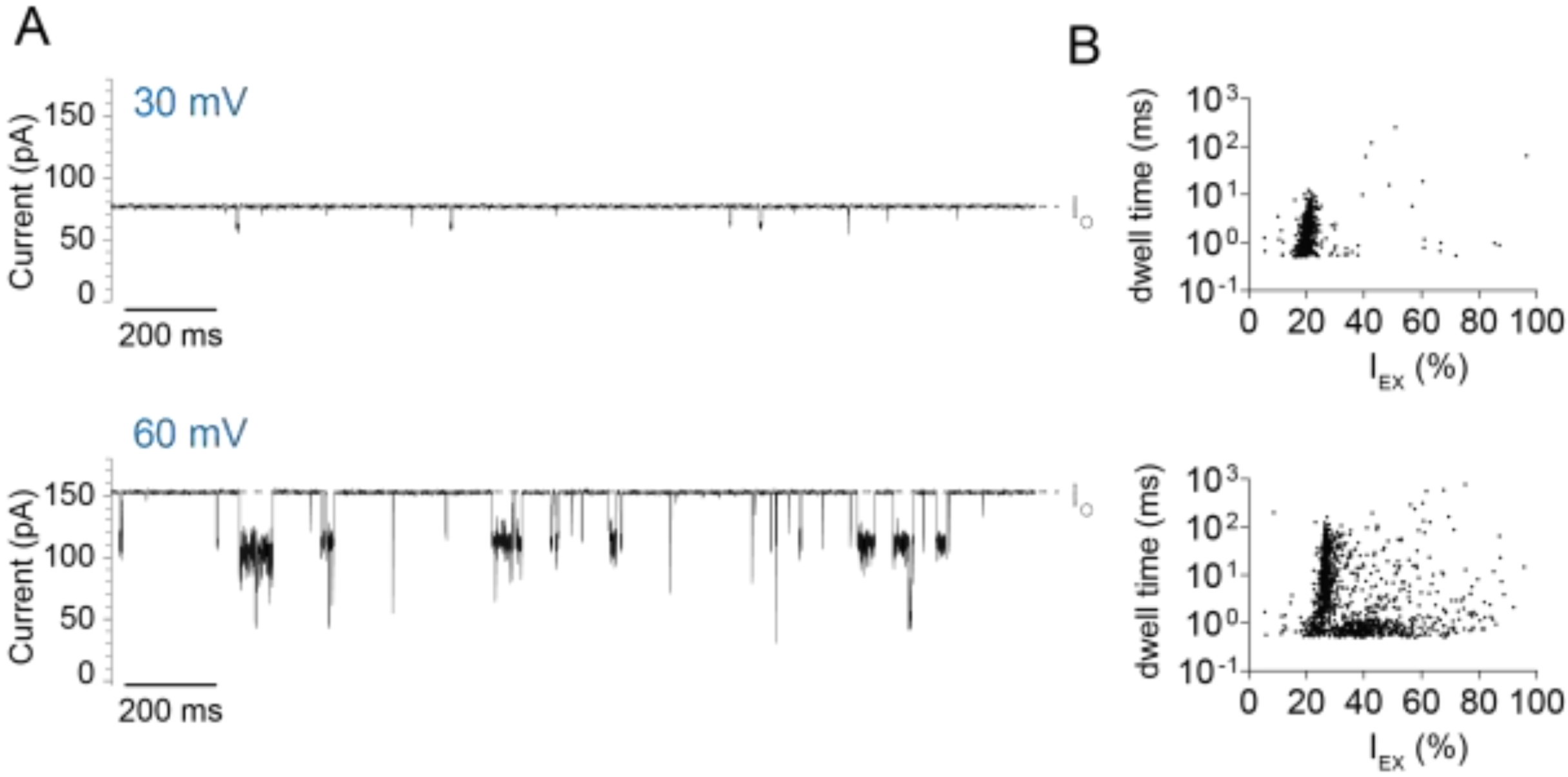

Supporting Figure 23: Reduced insulin detected by MspA-M2-S103K-I105K[2.6*]. A) Electrophysiology trace of MspA-M2-S103K-I105K capturing reduced insulin. Externally applied voltage is in blue. I_O is grey dotted line. **B)** Corresponding scatterplot with n>500 datapoints. Insulin (10 µM) was pre-incubated with 5 mM DTT, for 1 h at room temperature, before added to *cis* at 250 nM. Measurements were conducted in 1 M NaCl, 50 mM TrisHCl pH 7.5, with $PDB_{11}PEO_8$ composing the bilayer. Data were recorded at 50 kHz sampling rate, 10 kHz Bessel filter. Traces were additionally filtered with 2 kHz low-pass Gaussian filter for visualization and analysis. Experiments were executed at least in triplicate (N>3 pores).

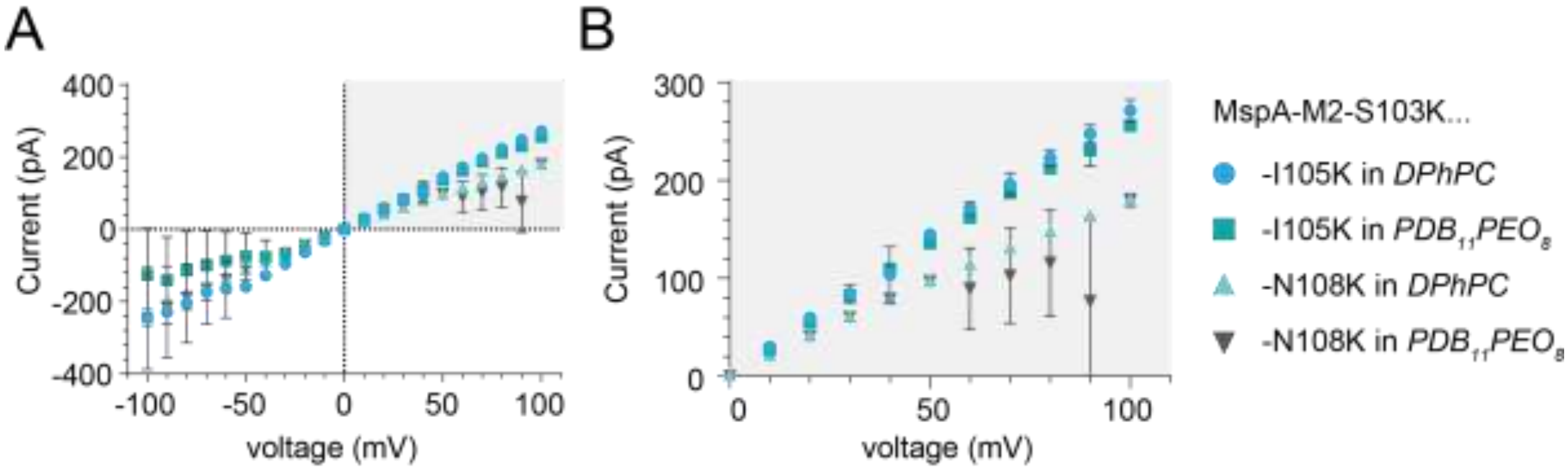

Supporting Figure 24: I/V curves of pores in different membrane compositions. A) I/V-curves of the respective MspA-M2-double mutants in the respective membrane. **B)** The zoom-in is indicated by the grey area. Measurements were conducted in 1 M NaCl, 50 mM TrisHCl pH 7.5. Data were recorded at 50 kHz sampling rate, 10 kHz Bessel filter. Traces were additionally filtered with 2 kHz low-pass Gaussian filter for visualization. Experiments were conducted at minimum in triplicate (N>3 pores). Error bars represent standard deviation.

References

(1) Baldelli, M.; Di Muccio, G.; Sauciuc, A.; Morozzo Della Rocca, B.; Viola, F.; Balme, S.; Bonini, A.; Maglia, G.; Chinappi, M. Controlling Electroosmosis in Nanopores Without Altering the Nanopore Sensing Region. *Advanced Materials* **2024**, *36* (33), 2401761. https://doi.org/10.1002/adma.202401761.

(2) A. Hatenboer-Sauciuc. Following the Tide–the Electroosmotic Transport of Proteins across Nanopores: Engineering, Biophysics and Potential Applications, PhD Thesis, Rijksuniversiteit Groningen, Groningen, 2025.

(3) Liaci, A. M.; Steigenberger, B.; Telles De Souza, P. C.; Tamara, S.; Gröllers-Mulderij, M.; Ogrissek, P.; Marrink, S. J.; Scheltema, R. A.; Förster, F. Structure of the Human Signal Peptidase Complex Reveals the Determinants for Signal Peptide Cleavage. *Molecular Cell* **2021**, *81* (19), 3934-3948.e11. https://doi.org/10.1016/j.molcel.2021.07.031.

(4) Rodriguez-Calvo, T.; Chen, Y.-C.; Verchere, C. B.; Haataja, L.; Arvan, P.; Leete, P.; Richardson, S. J.; Morgan, N. G.; Qian, W.-J.; Pugliese, A.; Atkinson, M.; Evans-Molina, C.; Sims, E. K. Altered β-Cell Prohormone Processing and Secretion in Type 1 Diabetes. *Diabetes* **2021**, *70* (5), 1038–1050. https://doi.org/10.2337/dbi20-0034.

(5) Waterhouse, A. M.; Procter, J. B.; Martin, D. M. A.; Clamp, M.; Barton, G. J. Jalview Version 2—a Multiple Sequence Alignment Editor and Analysis Workbench. *Bioinformatics* **2009**, *25* (9), 1189–1191. https://doi.org/10.1093/bioinformatics/btp033.
